# Loss of hepatocyte cell division leads to liver inflammation and fibrosis

**DOI:** 10.1371/journal.pgen.1009084

**Published:** 2020-11-04

**Authors:** Matthew R. Dewhurst, Jin Rong Ow, Gözde Zafer, Noémi K. M. van Hul, Heike Wollmann, Xavier Bisteau, David Brough, Hyungwon Choi, Philipp Kaldis

**Affiliations:** 1 Institute of Molecular and Cell Biology (IMCB), A*STAR (Agency for Science, Technology and Research), Singapore; 2 Lydia Becker Institute of Immunology and Inflammation; and Division of Neuroscience and Experimental Psychology, School of Biological Sciences, Faculty of Biology, Medicine and Health, Manchester Academic Health Science Centre, University of Manchester, Manchester, United Kingdom; 3 Department of Biochemistry, National University of Singapore (NUS), Singapore; 4 Department of Medicine, Yong Loo Lin School of Medicine, National University of Singapore, Singapore; 5 Department of Clinical Sciences, Lund University, Clinical Research Centre (CRC), Sweden; HudsonAlpha Institute for Biotechnology, UNITED STATES

## Abstract

The liver possesses a remarkable regenerative capacity based partly on the ability of hepatocytes to re-enter the cell cycle and divide to replace damaged cells. This capability is substantially reduced upon chronic damage, but it is not clear if this is a cause or consequence of liver disease. Here, we investigate whether blocking hepatocyte division using two different mouse models affects physiology as well as clinical liver manifestations like fibrosis and inflammation. We find that in P14 *Cdk1*^*Liv-/-*^ mice, where the division of hepatocytes is abolished, polyploidy, DNA damage, and increased p53 signaling are prevalent. *Cdk1*^*Liv-/-*^ mice display classical markers of liver damage two weeks after birth, including elevated ALT, ALP, and bilirubin levels, despite the lack of exogenous liver injury. Inflammation was further studied using cytokine arrays, unveiling elevated levels of CCL2, TIMP1, CXCL10, and IL1-Rn in *Cdk1*^*Liv-/-*^ liver, which resulted in increased numbers of monocytes. Ablation of CDK2-dependent DNA re-replication and polyploidy in *Cdk1*^*Liv-/-*^ mice reversed most of these phenotypes. Overall, our data indicate that blocking hepatocyte division induces biological processes driving the onset of the disease phenotype. It suggests that the decrease in hepatocyte division observed in liver disease may not only be a consequence of fibrosis and inflammation, but also a pathological cue.

## Author summary

“Pathological” polyploidy is a hallmark of liver diseases like NAFLD and NASH. Since loss of *Cdk1* induces polyploidy, we used a mouse model with a hepatocyte-specific deletion of *Cdk1* or *Ccna2* to study the immediate effects on liver physiology. To our surprise, impaired hepatocyte proliferation results in the development of inflammation and fibrosis in young animals despite the lack of any treatment to promote liver damage. Furthermore, we show that this could be caused by polyploidy, as concurrent loss of *Cdk2* reverses polyploidy and the inflammatory phenotype. Thus, we provide evidence that the loss of hepatocyte proliferation in liver disease is not only an outcome but could also be an etiology of liver pathology.

## Introduction

The liver, besides being the metabolic center, is also the main organ responsible for neutralizing toxic substances in the body, which results in functional parenchymal hepatocytes being constantly exposed to toxins that can induce cell death. To alleviate this, the liver exhibits an impressive ability to repair itself, a feat accomplished by the re-entry of fully differentiated hepatocytes into the cell cycle [1]. A study in rats estimated that 0.2% to 0.5% of hepatocytes undergo cell division at any one point of time to maintain homeostatic regeneration in normal adult non-diseased liver [2].

It is well-established that the ability of hepatocytes to undergo cell division upon induction of liver regeneration is impaired in models of severe liver disease [3–5]. This can be a result of replicative senescence caused by the continual cycling of hepatocytes upon chronic liver damage driven by lysophosphatidylcholine-mediated lipotoxicity [6] as is the case for hepatic steatosis, or inflammatory cytotoxicity induced by viral hepatitis [7, 8]. Notably, this is evidenced by shortened telomeres and increased senescence markers in the liver of patients with non-alcoholic fatty liver disease (NAFLD) and cirrhosis [9–11]. Alternatively, oxidative stress can also trigger the DNA damage checkpoint to inhibit proliferation and promote polyploidization in hepatocytes, a phenotype also seen in patients with non-alcoholic steatohepatitis (NASH) [12].

Progression through the different phases of the cell cycle is regulated by the activity of a family of kinases known as cyclin-dependent kinases (CDKs), named as such for the pre-requisite interaction with another family of proteins called cyclins in order to be functional [13]. CDK1, the first CDK to be identified, was initially discovered as part of the maturation-promoting factor that was crucial and sufficient for inducing mitosis [14]. The importance of CDK1 in driving mitosis, and more generally, cell proliferation, is exemplified by the observation that inhibition of CDK1 activity arrests cells in the G2 phase and prevents cell division [15, 16]. Accordingly, constitutive genetic ablation of *Cdk1* results in embryonic lethality [17]. On the other hand, CDK2, a paralog of CDK1, despite being responsible for promoting DNA replication in S phase [18], is not essential for viability because of functional compensation by CDK1 [19, 20] and CDK4. Both CDK1 and CDK2 partner with cyclin A2 (*Ccna2*) and cyclin B to reach full activity during S phase and mitosis.

Hepatocytes can exhibit an altered cell division process whereby mitosis occurs without the completion of cytokinesis, leading to the formation of binuclear polyploid cells [21]. This happens physiologically downstream of the insulin signaling pathway [22] and may serve to protect against loss of tumor suppressors upon genomic aberrations induced by a nutrient-rich environment [23, 24]. Interestingly, polyploidization involving mononuclear polyploid cells that do not complete mitosis, termed “pathological polyploidization”, has been observed in diseased liver of viral hepatitis and NAFLD patients [12, 25], implicating pathological polyploidization as a possible etiological factor in liver disease.

We and others have shown that genetically blocking cell cycle progression in hepatocytes without the exogenous induction of liver damage can, by itself, result in increased nuclear and cell diameter that is dependent on the state of ploidy [15, 26–29]. In particular, ablation of *Cdk1* specifically in the liver using Albumin-Cre (*Cdk1*^*Liv-/-*^) resulted in increased CDK2 activity leading to DNA re-replication and polyploidy [15]. Nevertheless, little else is known about the impact of this change in the hepatocyte phenotype on liver physiology and function.

Here, we aim to understand the implications of blocking hepatocyte cell division on liver physiology. Utilizing *Cdk1*^*Liv-/-*^ and *Ccna2*^*Liv-/-*^ mice as models of impaired hepatocyte proliferation, we observe the development of inflammation and fibrosis despite the lack of any treatment to promote liver damage. Furthermore, we show that this might be due to polyploidy, as concurrent loss of *Cdk2* leads to the absence of polyploidy reverting the phenotype. Thus, we provide evidence that the loss of hepatocyte proliferation in liver disease may not only be the outcome, but could also be an etiology of liver pathology.

## Results

### Liver-specific deletion of *Cdk1* begins embryonically and is complete by P14

“Pathological” polyploidy is often observed in liver diseases like NAFLD and NASH [12]. Since inhibition or loss of *Cdk1* is known to induce polyploidy [15], we used mouse models with a hepatocyte-specific deletion of *Cdk1* (*Cdk1*^*Liv-/-*^) or *Ccna2* (cyclin A2, see Fig 6). Hepatocytes undergo rapid mitotic division during early postnatal growth, however, upon maturation and terminal differentiation, readily quiesce [30]. To confirm *Cdk1* deletion and the loss of CDK1 protein, we chose two developmental windows when hepatic cells are actively undergoing division in which to observe *Cdk1* expression levels in *Cdk1*^*Liv-/-*^ animals–embryonic day 14.5 (E14.5) and postnatal day 14 (P14).

**Fig 1:**
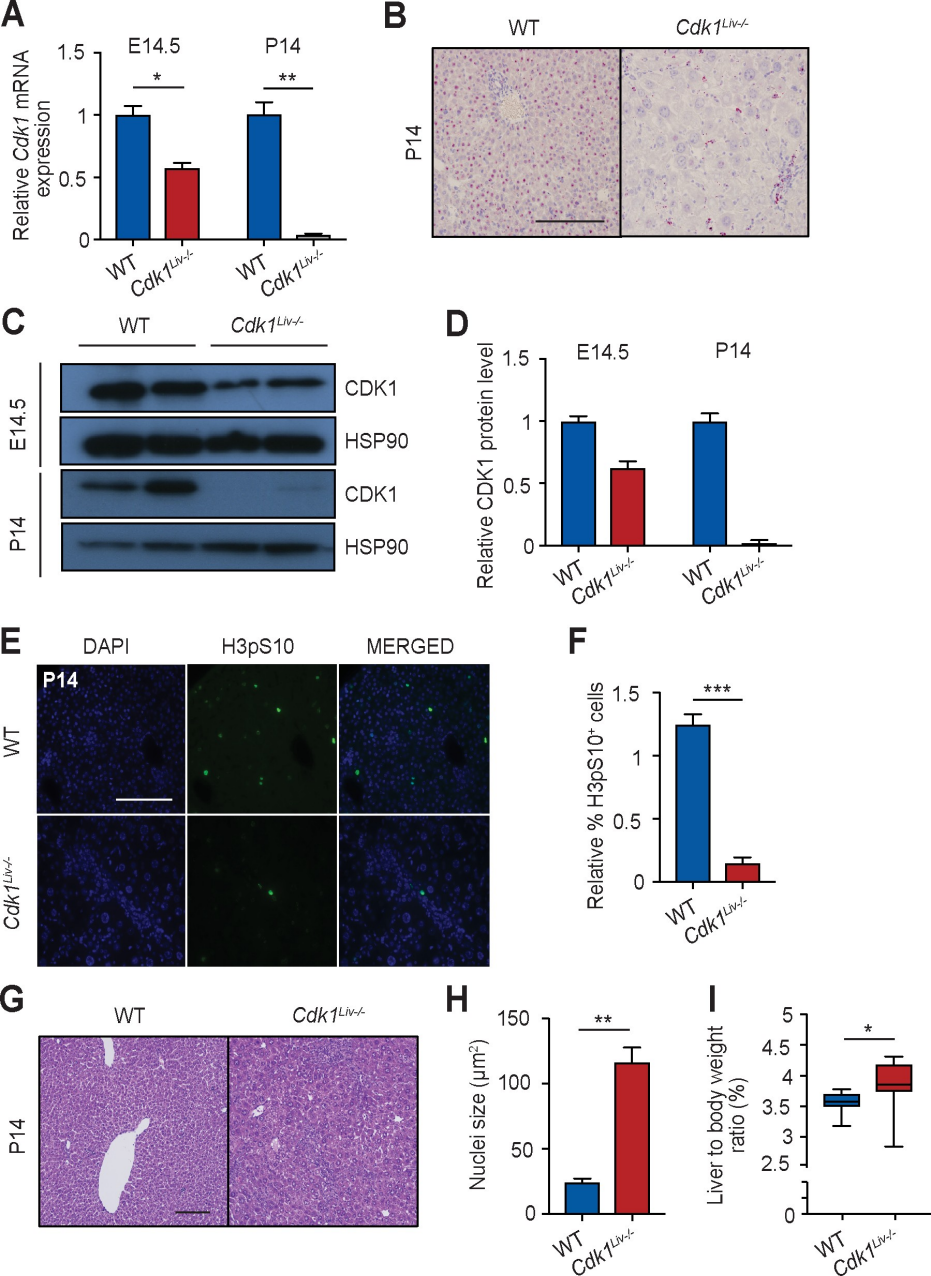
CDK1 is knocked-out by P14 in *Cdk1*^*Liv-/-*^ hepatocytes. (A) qPCR for *Cdk1* mRNA expression in pre-natal hepatoblasts (E14.5) and post-natal hepatocytes (P14) (n = 3 mice per genotype per time point). (B) Representative image of *in situ* hybridization for exon 3 of *Cdk1* on liver sections using the BaseScope assay. (C) Immunoblot of lysates from hepatoblasts (E14.5) and hepatocytes (P14) probed for CDK1 with HSP90 as loading control. (D) Quantification of CDK1 protein level from western blot, normalized to HSP90 protein levels. Error bars represent S.D. (E) Representative immunofluorescence images of sections stained for DAPI (blue), denoting the nuclei, and histone H3 pS10 (green), a marker for mitosis. (F) Quantification of H3pS10^+^ cells using at least 3,000 hepatocytes per mouse (n = 3 per genotype). Error bars represent S.E.M. (G) Representative H&E images of liver sections from P14 hepatocytes. (H) Quantification of nuclei size of P14 hepatocytes using at least 200 hepatocytes per mouse (n = 3 per genotype). (I) Ratio of liver weight to body weight, shown as percentage (%) of P14 mice (at least n = 8 per genotype). Error bars represent S.E.M. unless otherwise indicated. Scale bar of all microscopy images represent 50μm.

We isolated hepatoblasts from E14.5 embryos and hepatocytes from P14 pups for analysis of mRNA and protein expression from *Cdk1*^*Liv-/-*^ and wild type (WT). In E14.5 *Cdk1*^*Liv-/-*^ hepatoblasts, *Cdk1* mRNA expression was reduced by 40% in comparison to WT, whereas in P14 hepatocytes, *Cdk1* mRNA expression could hardly be detected (Fig 1A). *In situ* hybridization for exon 3 of *Cdk1* mRNA, which is the region on the *Cdk1*^*FLOX*^ allele [15] removed by Cre recombinase activity, resulted in intense staining in WT P14 hepatocytes, while staining in *Cdk1*^*Liv-/-*^ liver was restricted to interstitial cells, leaving hepatocytes unstained (Fig 1B). Western blotting unveiled that the CDK1 protein was expressed in WT E14.5 hepatoblasts and P14 hepatocytes (Fig 1C). We were able to detect a 40% reduction in CDK1 protein levels in *Cdk1*^*Liv-/-*^ hepatoblasts compared to WT (Fig 1D). At P14, CDK1 protein could not be detected in *Cdk1*^*Liv-/-*^ hepatocytes, suggesting that *Cdk1* was completely deleted by this stage. In summary, Albumin-Cre leads to partial deletion of *Cdk1* in embryonic hepatoblasts and complete deletion in hepatocytes two weeks after birth.

### Deletion of *Cdk1* prevents hepatocyte mitotic division

CDK1 drives mitosis in eukaryotic cells and is an essential gene in the mouse [15, 17]. To determine whether deletion of *Cdk1* was indeed impairing hepatocyte mitotic division *in vivo* at P14, liver sections were stained for phospho-serine 10 on histone H3 (H3pS10), a classical marker of mitosis [31]. Only background staining of H3pS10 was observed in *Cdk1*^*Liv-/-*^ livers compared to WT livers (Fig 1E and 1F), indicating that deletion of *Cdk1* was preventing P14 hepatocytes from undergoing mitotic division, similar as we have shown in adult hepatocytes [32].

### Loss of cell division induces hepatocyte hypertrophy and polyploidy

Since *Cdk1*^*Liv-/-*^ hepatocytes are unable to divide, it was surprising to observe that these animals developed a seemingly functional liver. To elucidate possible mechanisms by which mitotically impaired hepatocytes could still maintain liver growth, we performed histopathological analyses of liver sections. A significant enlargement of hepatocyte nuclei and an increase in hepatocyte size was observed during postnatal development (Fig 1G and 1H), which was consistent with previous literature [15]. In addition, the liver to body weight ratio was slightly but consistently increased in *Cdk1*^*Liv-/-*^ mice (Fig 1I). Overall, these results indicate that despite hepatocytes being unable to divide in *Cdk1*^*Liv-/-*^ mice, they do develop a liver that is at least as large as WT mice.

**Fig 2:**
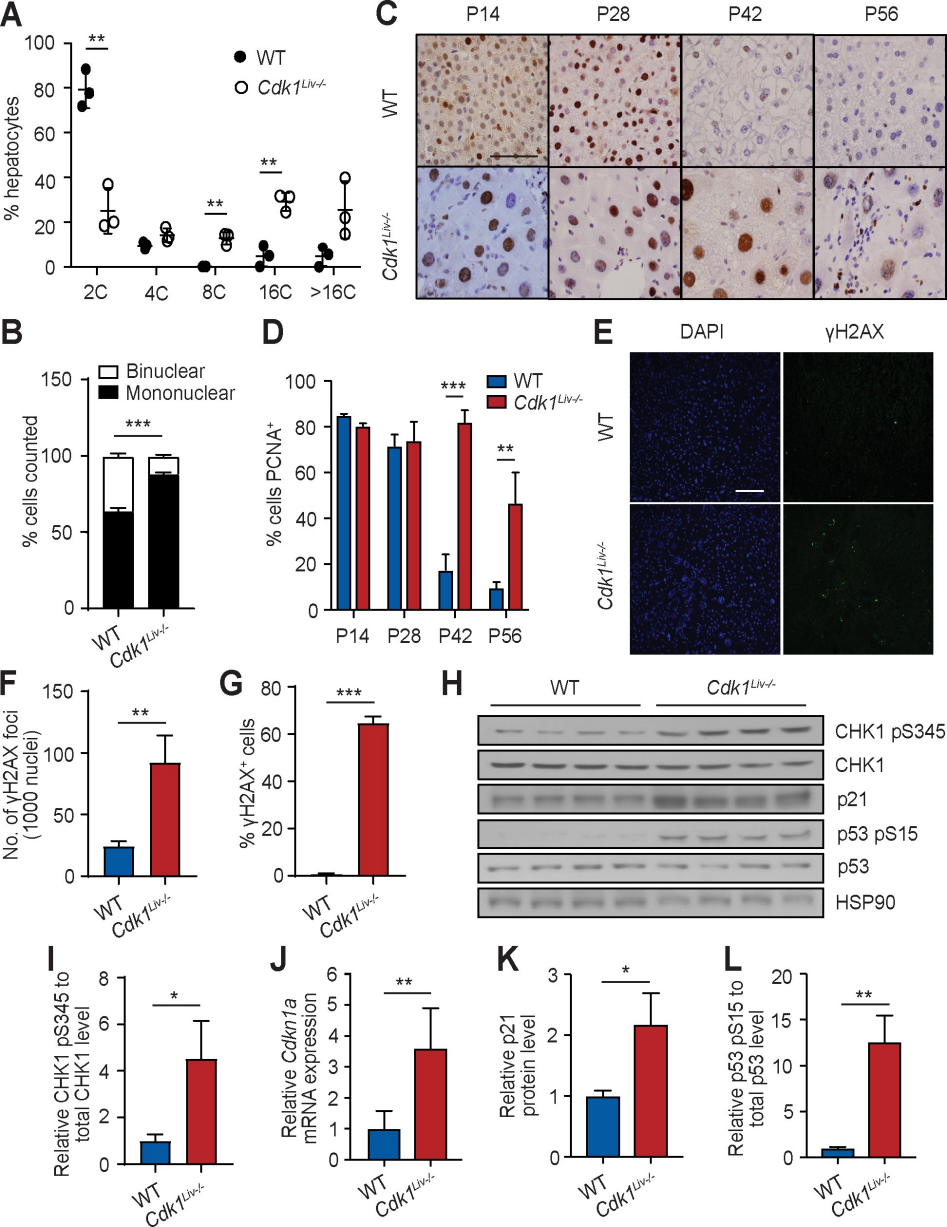
*Cdk1*^*Liv-/-*^ hepatocytes do not proliferate but undergo DNA re-replication. (A) DNA content analysis by flow cytometry of propidium iodide-stained hepatocytes isolated at P14. (B) Quantification of binuclear and mononuclear hepatocytes using at least 3,000 hepatocytes per mouse (at least n = 4 per genotype). (C) Representative immunohistochemistry images of sections stained for PCNA (brown), a marker for DNA replication, at different post-natal time points. (D) Quantification of PCNA^+^ cells using at least 200 hepatocytes per mouse (n = 5 per genotype per time point). (E) Representative immunofluorescence images of sections stained for DAPI (blue), denoting the nuclei, and γH2AX (green), a marker for DNA damage. (F) Quantification of γH2AX foci from 1000 nuclei using at least 5 images per mouse (at least n = 3 per genotype). (G) Quantification of γH2AX^+^ cells using at least 1,500 hepatocytes per mouse (at least n = 3 per genotype). (H) Immunoblot of lysates from P56 whole liver probed for CHK1 pS345, total CHK1, p21^Cip1/Waf1^, p53 pS15 and total p53, using HSP90 as loading control. (I) Quantification of ratio of CHK1 pS345 to total CHK1 levels. (J) qPCR for *Cdkn1a* mRNA expression in P56 whole liver sample (n = 6 per genotype). Quantification of p21 protein levels (K) and the ratio of p53 pS15 to total p53 levels (L). Error bars of all graphs excluding immunoblot quantification represent S.E.M. Error bars of immunoblot quantification represent S.D. Scale bar of all microscopy images represent 50μm.

It was previously reported that mitotically-impaired fibroblasts undergo cycles of complete DNA re-replication to generate polyploid cells [15]. FACS analysis of propidium iodide stained hepatocytes isolated from P14 animals was performed to determine whether a similar mechanism was occurring during early postnatal growth. WT hepatocytes were mostly diploid [80%] but a minority was polyploid [20%] (Fig 2A). Hepatocytes isolated from P14 *Cdk1*^*Liv-/-*^ animals displayed an abnormal pattern of DNA content with only ≈30% being diploid and the rest being polyploid compared to wild type (Fig 2A). Notably, we observed more mononuclear polyploid hepatocytes in *Cdk1*^*Liv-/-*^ than WT liver, and correspondingly, less binuclear hepatocytes (Fig 2B), indicating an overall increase in mononuclear polyploid hepatocytes due to a block in nuclear division [32]. To understand the increase in polyploidy, we stained liver sections for PCNA, a marker of DNA replication [33]. At P14 and P28, approximately 70–80% of the hepatocytes were positive for PCNA in both WT and *Cdk1*^*Liv-/-*^ (Fig 2C and 2D). In WT, hepatocytes ceased to replicate their DNA around P42-56 with less than 10% positive PCNA cells since most adult hepatocytes become quiescent at that stage. In contrast, *Cdk1*^*Liv-/-*^ hepatocytes maintained a high level of PCNA staining with approximately 80% positive for P42, and approximately 50% positive for PCNA at P56. This would suggest that *Cdk1*^*Liv-/-*^ hepatocytes might either continue to replicate DNA after control hepatocytes had entered into a quiescent state, or remain arrested in S phase unable to exit the cell cycle in the absence of CDK1. Given that cell size and DNA content are positively correlated [34], our data suggest that polyploidy-induced hypertrophy of hepatocytes drives postnatal liver growth in the absence of CDK1, which is in agreement to what we have reported for liver regeneration [32].

It has previously been shown that DNA re-replication and polyploidy can lead to a DNA damage response [35]. γH2AX foci, as a marker for DNA damage, could hardly been detected in WT P56 liver sections (Fig 2E) and were completely absent at P14. On the other hand, in P56 *Cdk1*^*Liv-/-*^ liver sections, there was a four-fold increase in γH2AX foci compared to WT (Fig 2F) with a corresponding increase in the percentage of γH2AX^+^ cells (Fig 2G). In addition, phosphorylated CHK1 at serine 345 (CHK1 pS345), a marker of DNA damage response [36], was increased in *Cdk1*^*Liv-/-*^ liver (Fig 2H and 2I). The expression of p21^*Cdkn1a*^ was found to be increased at both the mRNA (Fig 2J) and protein level (Fig 2H and 2K) in P56 *Cdk1*^*Liv-/-*^ compared to wild type liver, likely caused by increased p53 activity as evident by a higher level of phosphorylation on serine15 [37] (p53 pS15; Fig 2H and 2L).

Sustained activation of p53 can lead to induction of apoptosis [38]. In isolated *Cdk1*^*Liv-/-*^ hepatocytes, we detected increased expression of pro-apoptotic genes *Bcl2l11* and *Bbc3* (S1A Fig), commonly known as *Bim* and *Puma*, respectively, both of which are established p53 targets [39–41]. Furthermore, through mitochondria-cytoplasm fractionation, we identified elevated levels of cytoplasmic cytochrome c (CYC) in *Cdk1*^*Liv-/-*^ hepatocytes (S1B and S1C Fig), suggesting there was increased release of mitochondrial cytochrome c into the cytoplasm, a key event in the apoptosis pathway [42]. In addition, there was an increased sub-G1 population of cells when we analysed our flow cytometry data (S1D Fig). Taken together, our data suggest that in proliferation-defective hepatocytes lacking CDK1, DNA damage (γH2AX) was induced, which triggered a DNA damage response through activation of p53, resulting in the increased incidence of apoptosis and other phenotypes (see below).

### Liver specific deletion of *Cdk1* promotes inflammation and liver damage

**Fig 3:**
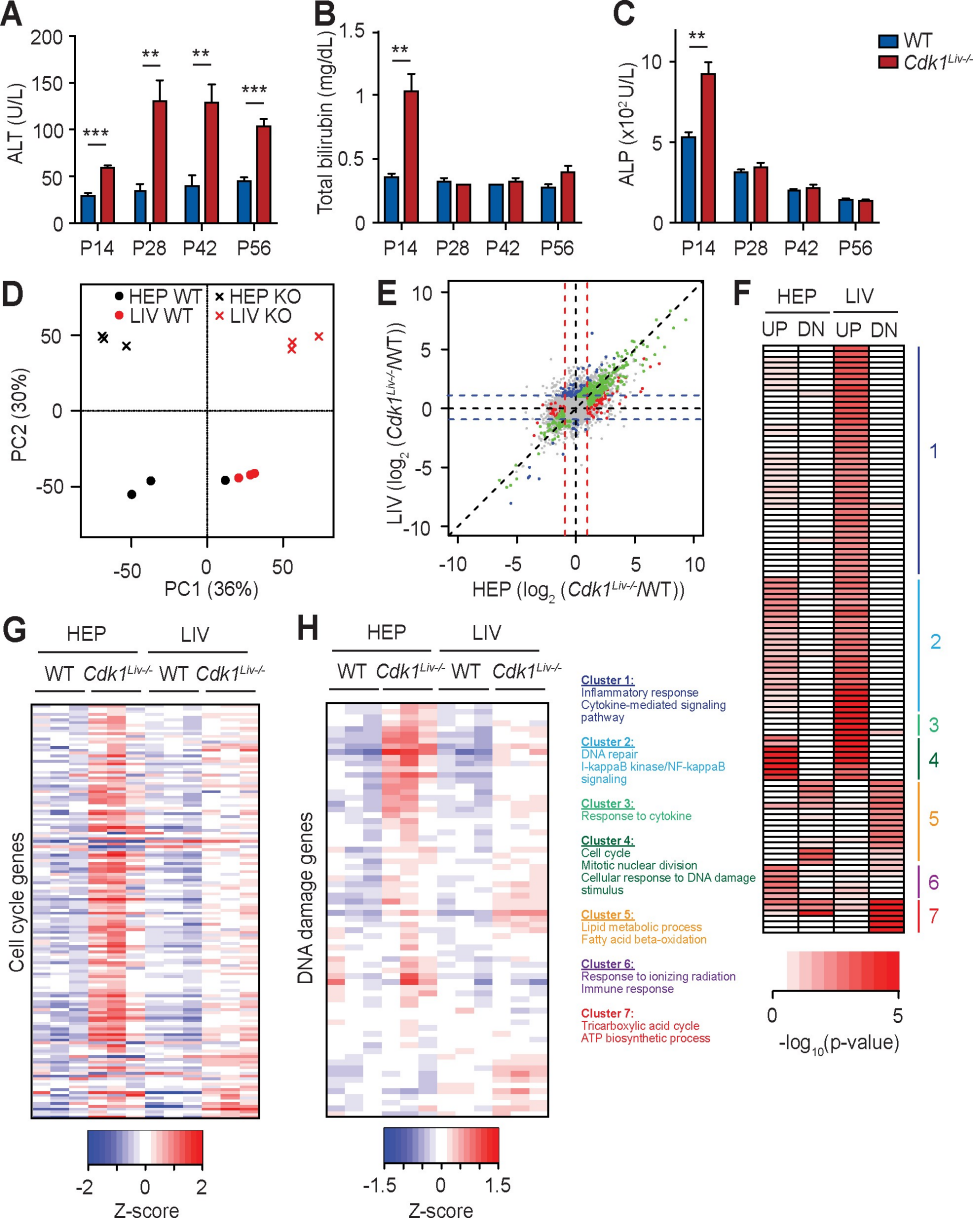
Transcriptomic analysis of *Cdk1*^*Liv-/-*^ hepatocytes and whole liver. Blood test for (A) alanine aminotransferase (ALT), (B) total bilirubin, and (C) alkaline phosphatase (ALP) levels at different post-natal time points (at least n = 5 per genotype per time point). (D) Principal component analysis of RNAseq results, with each dot or cross representing one sample that is either whole liver (LIV) or isolated hepatocyte (HEP) from wild type (WT) or *Cdk1*^*Liv-/-*^ (KO) mice. (E) Correlation plot of RNAseq results from whole liver and isolated hepatocytes, with each dot representing one gene that is either not significantly differentially expressed (grey), significantly differentially expressed in whole liver only (blue), in isolated hepatocytes only (red), or in both whole liver and isolated hepatocytes (green). (F) Heat map of gene ontologies of genes up-regulated (UP) or down-regulated (DN) in *Cdk1*^*Liv-/-*^ compared to WT samples with representative gene ontologies from each cluster listed. Color scale represents–log_10_(p-value). (G) Heat map of cell cycle associated genes from RNAseq analysis. (H) Heat map of DNA damage associated genes from RNAseq analysis. Color scales represent Z-score unless otherwise specified. Error bars of all graphs represent S.E.M.

Despite *Cdk1*^*Liv-/-*^ hepatocytes maintaining control of the liver-to-body weight ratio (Fig 1I), markers of hepatocyte damage were observed (Fig 2). Therefore, we aimed to analyse the physiological parameters of these mice. In WT mice, ALT (blood alanine transaminase) and bilirubin levels were constant (Fig 3A and 3B), whereas ALP (alkaline phosphatase) levels decreased from 500 U/L at P14 to 150 U/L at P56 (Fig 3C). In *Cdk1*^*Liv-/-*^ liver, ALT was already elevated at P14 and was four-fold higher after P28 (Fig 3A). Bilirubin and ALP levels were increased at least two-fold at P14 in *Cdk1*^*Liv-/-*^ compared to WT, but at later time points, *Cdk1*^*Liv-/-*^ and WT were similar (Fig 3B and 3C). These data indicated that there was initial injury at P14, which seemed to affect ALT at the later time points. Although this could be a result of DNA damage (see Fig 2), one could envision alternative causes too.

To understand the cause of liver damage, we performed transcriptomic analysis of isolated hepatocytes and whole liver from WT and *Cdk1*^*Liv-/-*^ mice at P14. We detected transcripts from 21,066 genes, of which 341 and 74 were respectively up-regulated and down-regulated in *Cdk1*^*Liv-/-*^ isolated hepatocytes. 392 and 59 were respectively up-regulated and down-regulated in *Cdk1*^*Liv-/-*^ whole liver when compared to WT (S1 Table). Principal component analysis (PCA) of the data indicated that the samples could generally be clustered according to CDK1 status and whether the sample was isolated hepatocytes or whole liver. Loss of CDK1 led to a shift of both isolated hepatocyte and whole liver samples along the PC2 axis in the same direction, suggesting that the change in transcriptomics upon *Cdk1* knockout was generally the same between hepatocytes and whole liver (Fig 3D). Globally, the majority of the genes remained expressed at the same levels in both *Cdk1*^*Liv-/-*^ and WT, indicating that only a minority of genes were significantly altered in the *Cdk1*^*Liv-/-*^ mice (Fig 3E). Gene ontology analysis was performed for differentially expressed genes (S2 Table), and genes up-regulated in *Cdk1*^*Liv-/-*^ hepatocytes were enriched for gene ontology terms associated with “mitotic nuclear division” and “cell cycle” (Fig 3F Cluster 4, S2 Fig). A closer look at genes involved in cell cycle regulation indicated that a majority of these genes were up-regulated in hepatocytes and to a lesser extent in the whole liver (Fig 3G, S3 Fig). As we observed a greater number of polyploid *Cdk1*^*Liv-/-*^ hepatocytes (Fig 2A), which might be caused by reduced cytokinesis [21], we also inspected for any changes in cytokinesis-associated genes. Similar to what was detected for other cell cycle-associated genes, the expressions of a number of cytokinesis-associated genes such as *Anln*, *Plk1*, and *Racgap1* were increased (S4 Fig). Notably, many cell cycle-related terms are enriched for genes up-regulated in *Cdk1*^*Liv-/-*^ whole liver as well (Fig 3F, S2 Fig), possibly because hepatocytes comprise up to 70% of all hepatic cells [43].

The enrichment of gene ontology terms such as “cellular response to DNA damage stimulus” (Fig 3F Cluster 2, S2 Fig) and “response to ionizing radiation” for up-regulated genes (Fig 3F Cluster 6, S2 Fig), coupled to the increase of many DNA damage response genes (Fig 3H, S5 Fig), implied involvement of DNA damage and p53 signalling in *Cdk1*^*Liv-/-*^ hepatocytes (see Fig 2H–2K). On the other hand, down-regulated genes were enriched for gene ontologies involved in “oxidation-reduction process” and “ATP biosynthetic process” (Fig 3F Cluster 7, S2 Fig), supporting our previous observation that *Cdk1*^*Liv-/-*^ hepatocytes are defective in oxidative phosphorylation [32]. Down-regulated genes were also enriched for “lipid metabolism” and “fatty acid oxidation” (Fig 3F Cluster 5, S2 Fig), which will be the focus of future studies.

**Fig 4:**
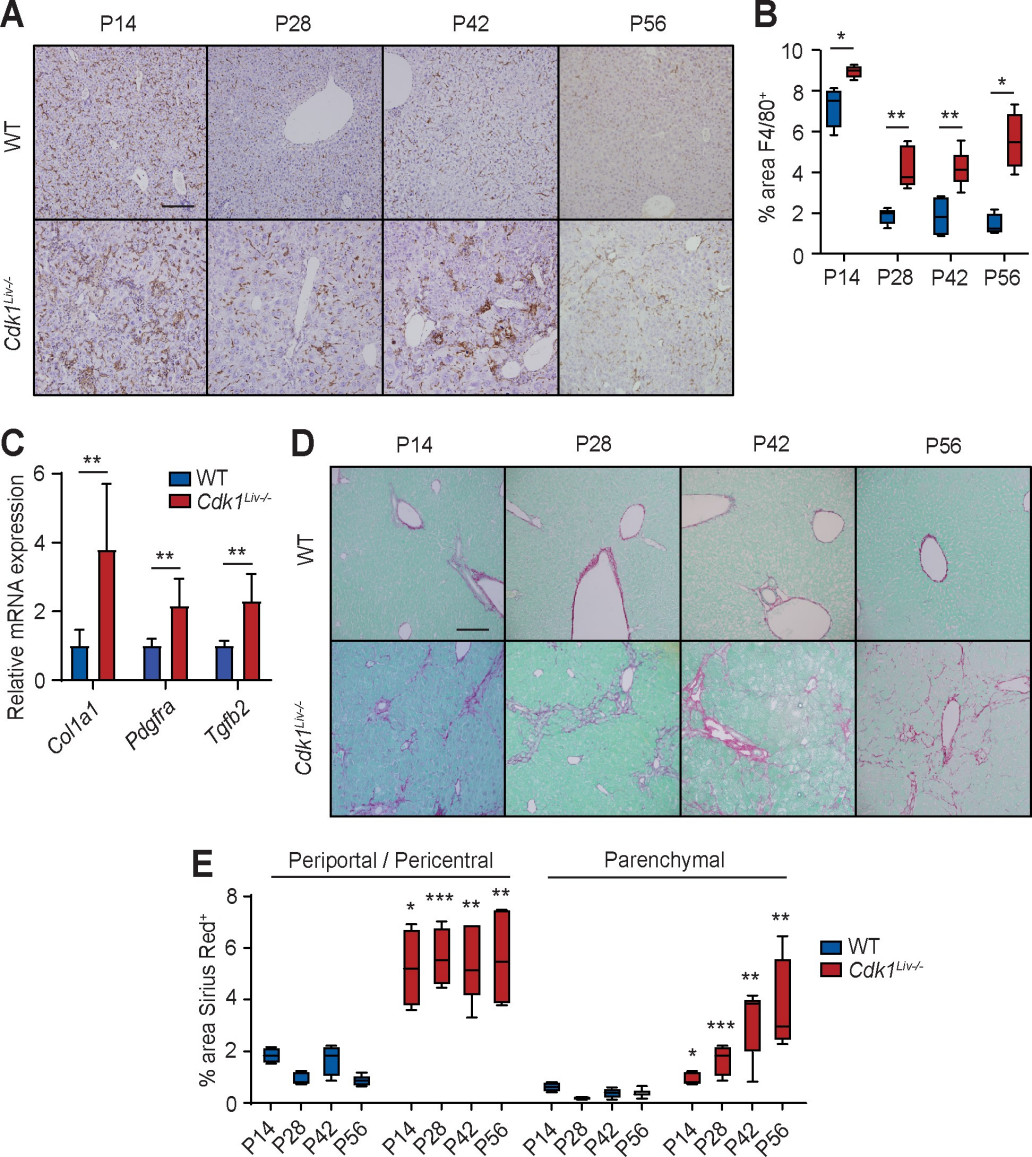
*Cdk1*^*Liv-/-*^ liver exhibits inflammation and fibrosis. (A) Representative immunohistochemistry images of sections stained for F4/80 (brown), a marker for macrophages, at different post-natal time points. (B) Quantification of F4/80^+^ cells using 5 images per mouse (at least n = 4 per genotype per time point). (C) qPCR for *Col1a1*, *Pdgfra*, and *Tgfb2* mRNA expression in whole liver sample (n = 6 per genotype). (D) Representative images of Sirius Red-stained liver sections at different post-natal time points. (E) Quantification of Sirius Red-stained area using at least 5 images per mouse (at least n = 4 per genotype per time point). Error bars of all graphs represent S.E.M. Scale bars of all microscopy images represent 50μm.

Chronic liver injury is often accompanied by pronounced hepatic inflammation and activation of the tissue resident macrophage population (Kupffer cells) [44]. The over-representation of genes involved in gene ontologies such as “inflammatory response” (Fig 3F Cluster 1, S2 Fig), “positive regulation of I-kappaB kinase/NF-kappaB signalling” (Fig 3F Cluster 2) and “response to cytokine” (Fig 3F Cluster 3) indicated that there might be increased inflammation in the liver of *Cdk1*^*Liv-/-*^ mice. To detect inflammation and activated Kupffer cells, liver sections were stained for F4/80, a classical marker of macrophages [45]. In WT liver sections, individual F4/80^+^ cells were scattered throughout the parenchyma at P14 and there was a 4-fold reduction at P28, remaining at low levels from then on (Fig 4A and 4B). The presence of large amounts of F4/80^+^ cells at this time point is possibly because monocytes and macrophages were in the expansion phase prior to establishing the resident Kupffer cell population during this developmental window [46]. In the *Cdk1*^*Liv-/-*^ liver sections, the number of F4/80^+^ cells was similar to WT at P14. In contrast to the reduction in F4/80^+^ staining in WT at P28-P56 when the Kupffer cells exited the expansion phase, aggregated foci and elevated numbers of Kupffer cells were observed in *Cdk1*^*Liv-/-*^ livers, which remained high at approximately 5% compared to 2% seen in WT livers at P14. These data indicate the presence of an inflammatory trigger in the *Cdk1*^*Liv-/-*^ liver from P28 onwards that maintains the F4/80^+^ cells in an active state.

An early indicator of chronic liver damage is the aberrant deposition of extracellular matrix resulting in fibrosis [47]. Analysis of RNAseq data for a gene signature of fibrosis [48] revealed increased expression of many of these genes (S6 Fig). A number of markers for fibrosis which were increased in the RNAseq at P14, such as *Col1a2*, *Pdgfra*, and *Tgfb2*, were validated by quantitative real-time PCR (qPCR) (Fig 4C), hinting at the presence of fibrosis in *Cdk1*^*Liv-/-*^ liver. To investigate fibrosis, liver sections were stained with Sirius Red to detect collagen deposition, a major component of extracellular matrix. In WT, the levels of collagen and, therefore, fibrosis was low at all time points measured and was restricted to the periportal and pericentral areas as expected (Fig 4D). In contrast, Sirius Red staining remained elevated, with periportal and pericentral staining approximately 5-fold higher than in WT, and a general increase in parenchymal staining suggesting classic bridging formation between periportal regions (Fig 4E). This indicated that loss of CDK1 in the liver resulted in elevated levels of fibrosis in the absence of external insults as early as P14.

### Chemokine levels in the liver and hepatocytes

**Fig 5:**
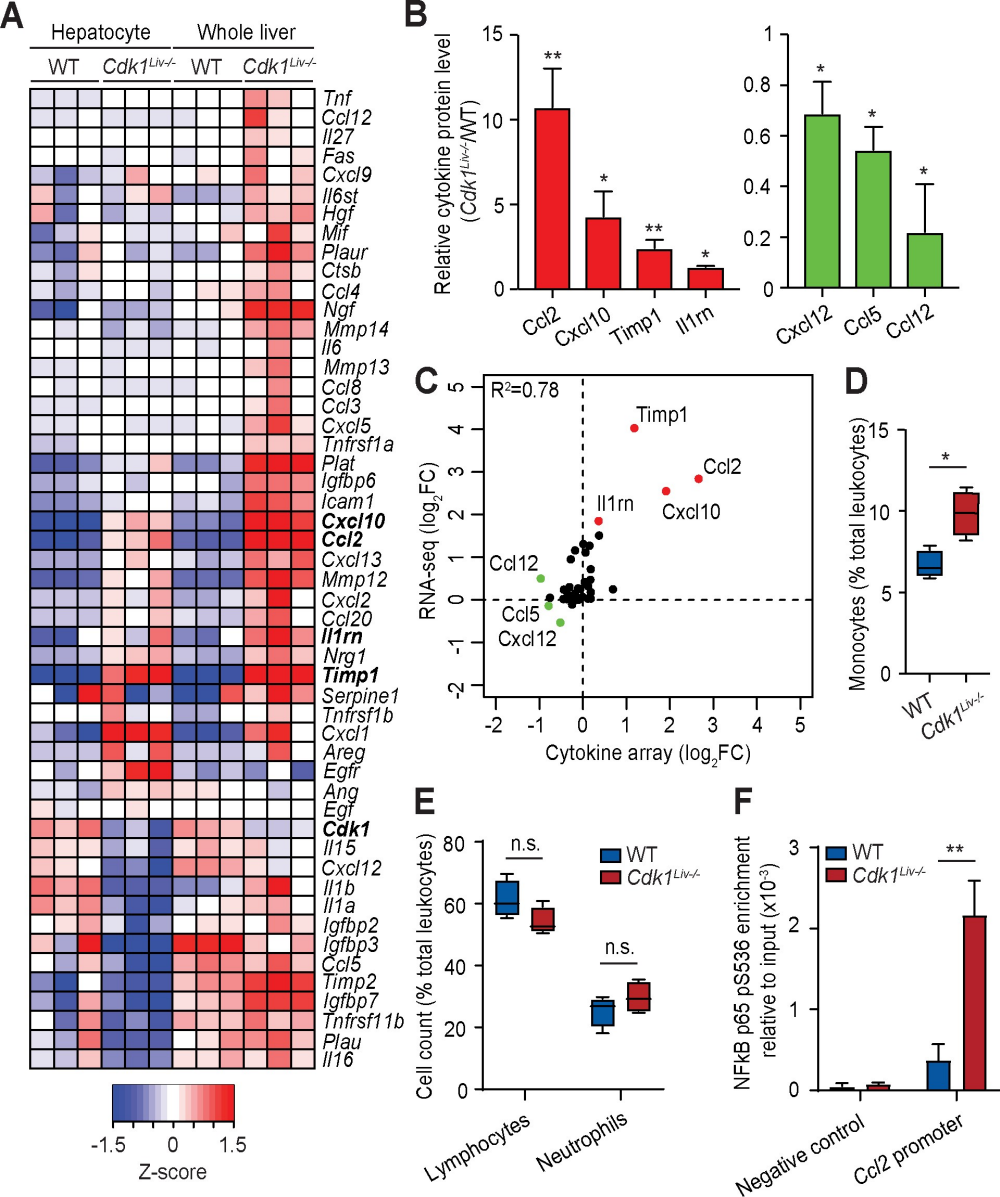
Characterization of the inflammatory response in *Cdk1*^*Liv-/-*^ liver. (A) Heat map of inflammation-associated genes from the RNAseq analysis. Color scale represents Z-score. (B) Cytokine array of lysates from P14 whole liver (n = 3 per genotype). (C) Correlation plot of cytokine levels from the RNAseq analysis and the cytokine array. Whole blood from wild type (WT) and *Cdk1*^*Liv-/-*^ mice were analyzed for (D) monocytes, (E) lymphocytes and neutrophils and shown as a percentage (%) of the total number of leukocytes analyzed from the same mouse (n = 4 per genotype). (F) ChIP for NFκB p65 pS536 from lysates of isolated hepatocytes, probing for the *Ccl2* promoter by qPCR (n = 3 per genotype). Error bars represent S.E.M.

Cells with DNA damage secrete a complex secretome that modulates both the immune cell milieu as well as the surrounding microenvironment [49]. To analyse this proinflammatory effect further, we screened our RNAseq data for inflammatory genes that were differentially expressed between WT and *Cdk1*^*Liv-/-*^ hepatocytes and whole liver. As expected, many cytokines and inflammatory-related genes were up-regulated in the liver of *Cdk1*^*Liv-/-*^ (Fig 5A). To further explore how this translated into a difference in the secretome, a cytokine protein array was performed on whole liver lysates. We detected elevated levels of CCL2, CXCL10, IL-1Rn, and TIMP1 as well as decreased levels of CXCL12 in *Cdk1*^*Liv-/-*^ livers compared to WT livers (Fig 5B), which were also the most differentially regulated in the whole liver transcriptomics (Fig 5C). Notably, *Ccl2*, *Timp1*, *Cxcl10*, and *Il1rn* mRNA levels were also increased in isolated *Cdk1*^*Liv-/-*^ hepatocytes, suggesting a contributory role of *Cdk1*^*Liv-/-*^ hepatocytes to the immune response.

CCL2 is a chemokine that promotes emigration of monocytes from the bone marrow into the circulation and their recruitment to sites of inflammation [50, 51]. Parallel to the rise in CCL2 levels, we detected a specific increase in circulating monocytes in the blood (Fig 5D), but not lymphocytes or neutrophils (Fig 5E). Elevated monocyte infiltration into the liver induced by CCL2 might, in part, explain the increase of F4/80^+^ cells (see Fig 4A and 4B).

CCL2 expression can be directly driven by NFκB, with the p65 subunit binding to the promoter of the *Ccl2* gene [52]. Since p65 can be phosphorylated at Ser 536 downstream of active p53 to promote NFκB-dependent transcription [53], we performed chromatin immunoprecipitation (ChIP) to test whether there was greater binding of p65 to the *Ccl2* promoter. Indeed, in *Cdk1*^*Liv-/-*^ hepatocytes, there was greater localization of NFκB p65 phosphorylated at serine 536 (NFκB p65 pS536) at the *Ccl2* promoter (Fig 5F). This suggests that the increase in CCL2 might be due to enhanced NFκB signaling, which might in turn be activated by greater p53 activity (Fig 2). In support, “I-kappaB kinase/NF-kappaB signalling” (Fig 3F Cluster 2) appears as an enriched gene ontology for up-regulated genes in not only whole liver transcriptomics, but also in isolated hepatocytes (Fig 3F).

**Fig 6:**
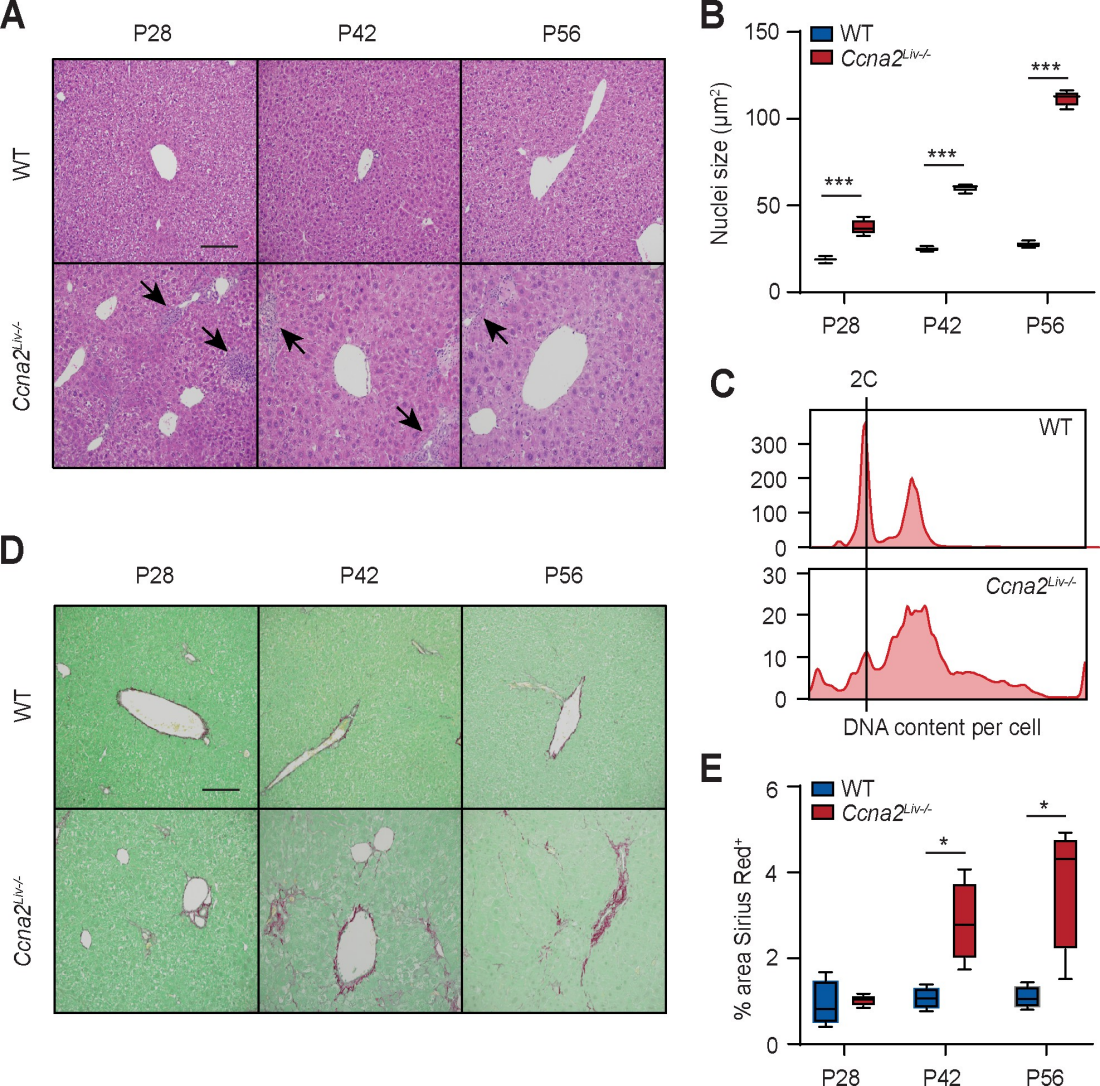
*Ccna2*^*Liv-/-*^ liver exhibits hypertrophy and fibrosis. (A) Representative H&E images of liver sections. Arrows point to areas of immune infiltration. (B) Quantification of nuclei size of hepatocytes using at least 500 hepatocytes per mouse (n = 4 per genotype per time point). (C) DNA content analysis by flow cytometry of propidium iodide-stained hepatocytes isolated from wild type (WT) and *Ccna2*^*Liv-/-*^ mice at P84. (D) Representative images of Sirius Red-stained liver sections. (E) Quantification of Sirius Red-stained area using at least 10 images per mouse (n = 4 per genotype per time point). Error bars represent S.E.M.

To ensure that the phenotype we described is not only observed in *Cdk1*^*Liv-/-*^ mice, we checked for fibrosis in a different mouse model of impaired hepatocyte proliferation, the *Ccna2*^*Liv-/-*^ mice, in which *Ccna2* (cyclin A2), an activator of CDK activity, is deleted specifically in hepatocytes using Albumin-Cre. We had previously reported that knocking out *Ccna2* in the liver impairs proliferation of hepatic cells [54]. As seen in *Cdk1*^*Liv-/-*^ liver, *Ccna2*^*Liv-/-*^ hepatocytes exhibit increased size (Fig 6A and 6B), although the magnitude of size increase was not as much as *Cdk1*^*Liv-/-*^ hepatocytes with the increase occurring slower and later in post-natal development. This is supported by flow cytometry analysis of DNA content in isolated hepatocytes (Fig 6C), which revealed significantly reduced percentage of cells with 2C content and greater percentage of cells >2C content. Unexpectedly, unlike *Cdk1*^*Liv-/-*^ hepatocytes, which exhibited distinct peaks that likely correspond to complete genome replication albeit being in a polyploid state, *Ccna2*^*Liv-/-*^ hepatocytes exhibit a DNA content profile resembling aneuploidy exacerbated by DNA re-replication. This might be due to the importance of cyclin A2 in maintaining DNA integrity during DNA replication and chromosome segregation [55]. Nevertheless, obvious areas of immune infiltration were also observed in H&E sections (Fig 6A, arrows), and when we probed sections of *Ccna2*^*Liv-/-*^ liver for fibrosis, we noted increased fibrosis from P42 onwards (Fig 6D and 6E). This hints at the presence of liver injury in *Ccna2*^*Liv-/-*^ mice even though no exogenous liver injury was applied and suggests that the inflammatory and fibrotic phenotype observed in *Cdk1*^*Liv-/-*^ liver may be relevant in other cases where hepatocyte proliferation is impaired.

### Blocking DNA replication prevents inflammatory and fibrotic phenotype

**Fig 7:**
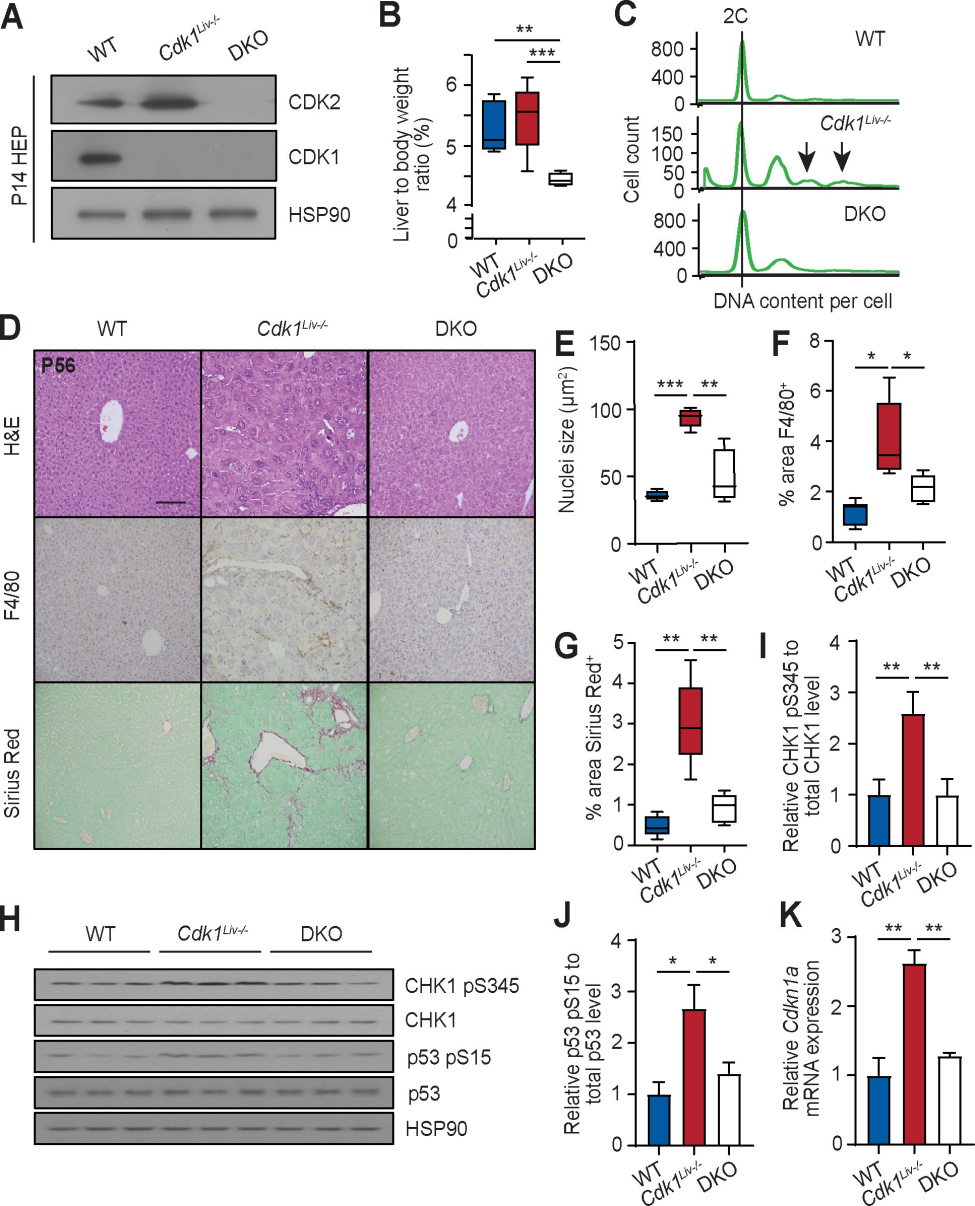
*Cdk2* knockout rescues phenotype of *Cdk1*^*Liv-/-*^ liver. (A) Immunoblot of lysates from P14 hepatocytes isolated from wild type (WT), *Cdk1*^*Liv-/-*^ and double knockout (DKO) mice, probed for CDK1 and CDK2 with HSP90 as loading control. (B) Ratio of liver weight to body weight, shown in percentage [%] (at least n = 4 per genotype). Error bars represent S.D. (C) DNA content analysis by flow cytometry of propidium iodide-stained hepatocytes isolated from wild type (WT), *Cdk1*^*Liv-/-*^, and double knockout (DKO) mice at P14. (D) Representative H&E images, immunohistochemistry images of sections stained for F4/80 (brown), and Sirius Red-stained images of liver sections. (E) Quantification of nuclei size of hepatocytes using at least 300 hepatocytes per mouse (n = 5 per genotype). (F) Quantification of F4/80^+^ area using at least 4 images per mouse (at least n = 5 per genotype). (G) Quantification of Sirius Red-stained area using at least 4 images per mouse (n = 5 per genotype). (H) Immunoblot of lysates from P14 isolated hepatocytes probed for CHK1 pS345, total CHK1, p53 pS15, and total p53, using HSP90 as loading control. Quantification of ratio of (I) CHK1 pS345 to total CHK1 levels and (J) p53 pS15 to total p53 levels. (K) qPCR for *Cdkn1a* mRNA expression in P14 isolated hepatocytes (n = 3 per genotype). Error bars of all graphs represent S.E.M. unless otherwise stated. Error bars of immunoblot quantification represent S.D. Scale bar of all microscopy images represent 50μm.

Hepatocytes are one of the rare differentiated cell types that exist as diploid as well as polyploid, but under normal conditions, polyploid cells are largely binuclear and the degree of polyploidy rarely exceeds 8C [56]. In contrast, in liver diseases like NAFLD and NASH, it was observed that the percentage of mononuclear polyploid cells and the degree of polyploidy is significantly increased, fueling the hypothesis that “pathological” polyploidy may be a driver of the disease [12]. In addition, polyploidy has been suggested to trigger a DNA damage response [57]. To understand whether “pathological” polyploidy contributes to the phenotypes observed in *Cdk1*^*Liv-/-*^ liver, we wanted to prevent DNA re-replication by removing the *Cdk2* gene in *Cdk1*^*Liv-/-*^ mice, creating compound knockout (DKO) mice. DKO mice were not born at Mendelian ratio in a *Cdk1*-null background (S3 Table). Nevertheless, we did not observe perinatal lethality, suggesting that embryonic lethality was a likely cause of DKO pups being born at lower-than-expected ratio. Fortunately, this phenotype was not completely penetrant and despite the loss of both CDK1 and CDK2 in the hepatocytes (Fig 7A), we were able to obtain DKO mice that survived, albeit with a lower liver to body weight ratio (Fig 7B).

Hepatocytes isolated from the DKO mouse did not exhibit polyploidy (Fig 7C, S7 Fig), confirming that CDK2 is indeed driving DNA re-replication in the *Cdk1*^*Liv-/-*^ liver *in vivo* as has been shown for MEFs *in vitro* [15]. While the level of ploidy in the *Cdk1*^*Liv-/-*^ hepatocytes at P14 is not as high as in adult mice (see Fig 2), we ascribe this mostly to a difference in the genetic background and age of these mice. Nevertheless, *Cdk1*^*Liv-/-*^ hepatocytes displayed a trend towards increased ploidy level, as seen by the higher 4C and the presence of 8C and 16C peaks indicated by arrows, with a correspondingly lower 2C peak even though equal numbers of cells were assessed and by P56, they exhibit the same phenotype as described earlier (see Fig 4). Interestingly, the size of DKO hepatocytes were larger than WT but not as large as *Cdk1*^*Liv-/-*^ hepatocytes (Fig 7D-E), supporting the idea that hypertrophy upon loss of hepatocyte division is partly dependent on polyploidy. Immunohistochemical staining for F4/80 as well as Sirius Red staining for fibrosis revealed reduced immune infiltration (Fig 7F) and fibrosis (Fig 7G) in DKO liver compared to *Cdk1*^*Liv-/-*^. When we probed the lysates of P14 isolated hepatocytes for markers of DNA damage (Fig 7H), we observed increased phosphorylation of CHK1 and p53 (Fig 7I and 7J) in *Cdk1*^*Liv-/-*^ hepatocytes, confirming what we described in P56 whole liver lysates (Fig 2) and implicating that DNA damage occurs as early as P14. In addition, we also noted that CHK1 and p53 phosphorylation was reduced in DKO hepatocytes to approximately the same level as WT hepatocytes (Fig 7I and 7J). Correspondingly, mRNA expression of *Cdkn1a* was also decreased back to WT levels in the absence of CDK2 (Fig 7K). This suggests that polyploidy or CDK2 itself has direct implications in promoting the DNA damage response, which in turn triggers the inflammatory phenotype observed in the liver of *Cdk1*^*Liv-/-*^ mice.

Overall, our data indicate that inhibiting hepatocyte division by deleting *Cdk1* or *Ccna2* right after birth leads to polyploidy that causes DNA damage early in development, which eventually results in inflammation that the damaged liver cannot resolve.

## Discussion

The ability of differentiated hepatocytes to re-enter the cell cycle is unique and is not only important for liver regeneration during acute injury (e.g. partial hepatectomy) but may also play a role in response to chronic injury. Here, it was our aim to investigate whether ablation of the hepatocyte self-renewal capacity would have any immediate consequences. Therefore, we focused on the analysis of liver two weeks after birth, a time point at which hepatocytes were still actively dividing, to understand the phenotypic changes in the absence of exogenous liver injury. A side effect of our approach of deleting the *Cdk1* gene was that *Cdk1*-deficient livers have significantly enlarged hepatocytes which is accompanied by increased polyploidy as we have described before [15, 32]. Therefore, we were also able to investigate the role of “pathological” hepatocyte polyploidy, which has been previously observed in liver disease. Finally, we were able to prevent the development of polyploidy by ablating CDK2 in the *Cdk1*^*Liv-/-*^ background (Fig 7C), which rescued the inflammation (Fig 7F) and decreased fibrosis (Fig 7G) in the double knockout liver (*Cdk1*^*Liv-/-*^*Cdk2*^*-/-*^).

Polyploidy occurs in normal human liver, with a majority of hepatocytes being binuclear tetraploids [56]. Physiologic polyploidy has been suggested to provide protection against genotoxic stress [58] and oncogenic loss of heterozygosity [23]. However, excessive polyploidy (>8C) and mononuclear polyploidy is increased in many cases of liver disease such as NAFLD/NASH [12] and viral hepatitis [25]. In our study, we have found that G2 phase-arrested *Cdk1*^*Liv-/-*^ hepatocytes undergo multiple rounds of DNA re-replication *in vivo* (Fig 1), eventually culminating in an elevation of octa- and higher levels of ploidy (Fig 2A). CDK1 kinase activity is essential for cell division and inhibition of CDK1 activity is sufficient to prevent mitotic progression [59] and block hepatic tumorigenesis [15]. Hence, despite the increase in cell cycle and cytokine-associated gene expressions (Fig 3G, S3 and S4 Figs), the loss of CDK1 kinase activity led to *Cdk1*^*Liv-/-*^ hepatocytes being unable to undergo mitosis (and correspondingly, nuclear division) as well as cytokinesis. This likely explains the presence of increased mononuclear polyploid hepatocytes, reflecting the occurrence of possible pathological polyploidization in *Cdk1*^*Liv-/-*^ liver.

The presence of pathological polyploidy could potentially activate the DNA damage response due to increased gene dosage of genes involved in the DNA damage pathway [57]. Additionally, excessive CDK2 activity in the absence of CDK1 leading to persistent DNA re-replication [15] can also trigger the DNA damage response due to increased stalled replication forks and exposure of single-stranded DNA at fragile sites in the genome [35, 60]. Indeed, our transcriptomic analysis (Fig 3), coupled to increased γH2AX, phosphorylated CHK1 and phosphorylated p53 (Fig 2), suggested the presence of DNA damage leading to activation of p53 and the DNA damage response in *Cdk1*^*Liv-/-*^ hepatocytes. With the absence of these phenotypes in the DKO mice (Fig 7C–7J), our study proposes a more direct role of DNA re-replication and polyploidy or CDK2 itself in these pathological processes.

The activation of p53 might be responsible for the increased incidence of apoptosis among isolated *Cdk1*^*Liv-/-*^ hepatocytes [38], shown by the elevated expression of pro-apoptotic genes and the cytoplasmic release of mitochondrial cytochrome c (S1 Fig), as well as the presence of a sub-G1 population of cells upon flow cytometry (Fig 7C, S1D Fig). Interestingly, a recent publication found that *TP53-*mutated hepatocellular carcinoma (HCC) is associated with pathological polyploidy, but not *Tert*- or *Ctnnb1*-mutated HCC [56]. While one can envision the development of pathological polyploidy in p53-deficient cells given the importance of p53 in the DNA damage response, from another angle, this observation agrees with our hypothesis. This is because only upon loss of p53 function would the development of pathological polyploidy in hepatocytes not induce p53-dependent apoptosis, thereby allowing transformation of hepatocytes to HCC.

**Fig 8:**
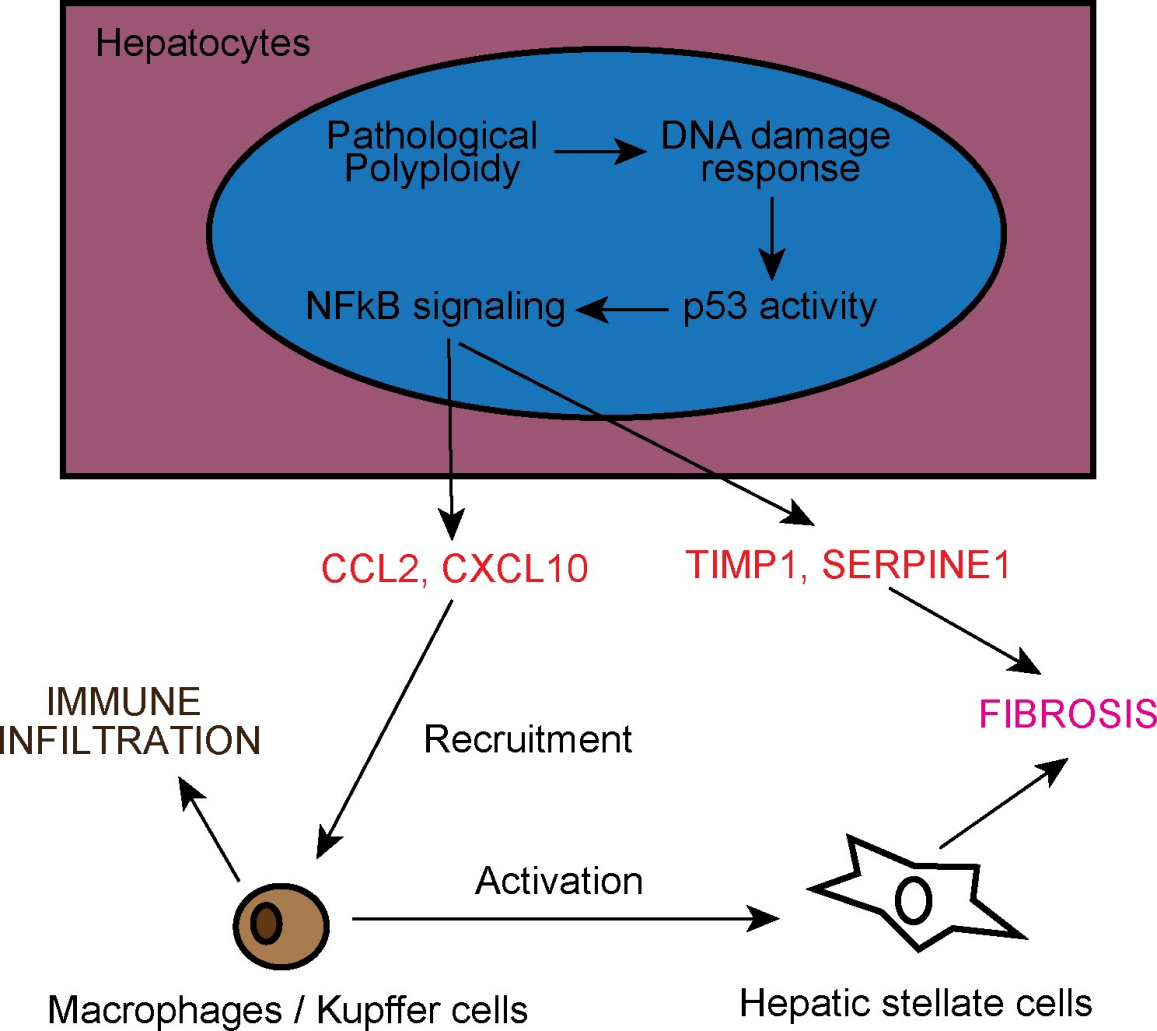
Model of liver inflammation and fibrosis in *Cdk1*^*Liv-/-*^ liver. Pathological polyploidy in *Cdk1*^*Liv-/-*^ hepatocytes activates p53 as part of the DNA damage response, which in turn triggers NFκB signaling. NFκB then induces expression of CCL2, and possibly CXCL10, SERPINE1, and TIMP1. CCL2 and CXCL10 recruit monocytes and macrophages leading to immune cell infiltration in the liver. Immune cells subsequently activate hepatic stellate cells to drive liver fibrosis. Separately, SERPINE1 and TIMP1 secreted by hepatocytes also promote the development of fibrosis by blocking the activity of proteases that breakdown extracellular matrix.

There is increased evidence linking inflammation with the progression of advanced liver diseases like NAFLD and NASH [61]. In the *Cdk1*^*Liv-/-*^ mouse model, we observe the presence of F4/80^+^ Kupffer cells starting at P14 and there was an early onset of inflammation in the absence of any external insult (Fig 4). This was possibly due to NFκB signalling triggered by p53 activity downstream of DNA damage (Fig 8). In particular, p53-dependent activation of NFκB acts via a non-canonical pathway involving the phosphorylation of NFκB p65 subunit by RPS6KA1 at S536 [53], although we cannot exclude the contribution from other signalling pathways given that S536 can be phosphorylated by a multitude of kinases [62]. NFκB p65 pS536 is then able to target genes such as *Ccl2* (Fig 5F), hence inducing increased expression of *Ccl2*. CCL2 can then promote increased immune infiltration of monocytes (Fig 4A and 4B) causing an inflammatory phenotype.

CCL2 is commonly associated with the pathogenesis of fibrosis. For example, increase in serum levels of CCL2 is associated with progression from hepatic steatosis to NASH [63], and CCL2 expression is correlated with disease severity in Hepatitis C-dependent liver fibrosis [64]. On the other hand, pharmacological inhibition of CCL2 accelerated fibrosis resolution in mouse models of liver fibrosis [65], thereby supporting the development of drugs targeting CCL2 receptors for NASH therapy [66]. As expected, with the increase in CCL2 levels, fibrosis was present in the *Cdk1*^*Liv-/-*^ liver, apparent by the up-regulation of fibrosis-associated genes (Fig 4C) and prevalent Sirius Red staining (Fig 4D). While most studies on fibrosis focus on the interactions between immune cells and hepatic stellate cells, our data suggest that hepatocytes are directly involved in promoting fibrosis (Fig 8) through up-regulation of *Ccl2* and *Cxcl10* to initiate inflammation, and also *Timp1* and *Serpine1* (Fig 5A), which are both important modulators of fibrosis [67, 68]. Interestingly, *Cxcl10*, *Timp1* and *Serpine1* are transcriptional targets of NFκB [69–71], further supporting the activation of NFκB in hepatocytes. In this respect, the *Cdk1*^*Liv-/-*^ mouse model is a useful model to study the commencing process of liver inflammation that leads to fibrosis and thereby more severe liver diseases.

It is noteworthy that we also observed hepatocyte hypertrophy and liver fibrosis in *Ccna2*^*Liv-/-*^ mice (Fig 6), a different mouse model of impaired hepatocyte proliferation [54]. Similarly, previous work on Survivin, a mitotic factor required for proper assembly and dissemination of metaphase chromosomes, indicated that the loss of Survivin in liver resulted in a block in mitosis that led to polyploidy, liver damage, and fibrosis [28]. This implies that our observations are not limited to only the *Cdk1*^*Liv-/-*^ mouse model, but might be a more generally applicable principle where hepatocytes are defective in proliferation. This also proposes that the mechanism of liver damage (Fig 8) in such cases is likely to be similar, and is worth looking at as a potential therapeutic avenue for ameliorating phenotypes in liver diseases that affect hepatocyte division.

In summary, we report here the analysis of a model where hepatocytes lack the capacity to divide. Interestingly, this model displays many of the hallmarks that are found in human patients with liver disease. Most strikingly, the phenotypes of this model develop without any external insults (like high fat diet and methionine/choline-deficient diet). In other words, all of the phenotypes in the *Cdk1*^*Liv-/-*^ mice develop spontaneously, which indicates that the loss of self-renewal capacity of hepatocytes has broad effects on inflammation, DNA damage, and fibrosis. Furthermore, we provide much-needed evidence implicating direct contribution of polyploidy to liver pathology. It remains to been seen whether similar effects can be detected in the human liver and how this can be exploited therapeutically.

## Materials and methods

### Ethics statement

Organs used in this study were obtained from mice housed and handled according to the National Advisory Committee for Laboratory Animal Research (NACLAR) Guidelines with all animal experiments approved by the A*STAR Institutional Animal Care and Use Committee (protocol #171268).

### Genetic mouse models, liver isolation and blood tests

*Cdk1*^*Liv-/-*^ (*Cdk1*^*flox/flox*^ Albumin-Cre) mice [15, 32] and *Ccna2*^*Liv-/-*^ (*Ccna2*^*flox/flox*^ Albumin-Cre) mice [54] have been described previously. Double knockout (DKO; *Cdk1*^*flox/flox*^ Albumin-Cre, *Cdk2*^*-/-*^) mice lacking both CDK1 and CDK2 in the liver were generated by crossing *Cdk1*^*Liv-/-*^ mice with *Cdk2*^*+/-*^ mice [72] and sibling mated for at least three generations before those with the right genotype were collected for tissue collection. Only male mice were used for experiments as to avoid hormonal effects as confounding factors. Mice were fed with standard diet *ab libitum* and housed in Specific Pathogen Free (SPF) conditions under 12-hour light/dark cycle at the A* STAR Biological Resource Center (A*STAR BRC) in Singapore.

### RNA extraction, chromatin immunoprecipitation (ChIP), and quantitative real-time PCR (qPCR)

RNA was extracted from isolated hepatocytes using the TRIzol reagent (15596018; Thermo Fisher Scientific) according to the manufacturer’s protocol. For snap frozen liver tissues, samples were homogenized in TRIzol reagent in a MP Biomedicals Lysing Matrix D tube (Thermo Fisher Scientific) using the Precellys 24 homogeniser (Bertin Technologies). 2μg of RNA was used to prepare complementary DNA (cDNA) using the Maxima First Strand cDNA Synthesis Kit (K1641; Thermo Fisher Scientific). ChIP was performed as described before [73] using 2μL of anti-NFκB p65 pS536 antibody (#3033; Cell Signaling Technology) per ChIP sample. DNA was then isolated from eluted samples using ChIP DNA Clean & Concentrator (D5205; Zymo Research) in 20μL volume. qPCR was then performed with the Maxima SYBR Green qPCR Master Mix (K0221; Thermo Fisher Scientific) using 10ng cDNA or 1uL of ChIP input/sample per reaction. Analysis was performed via the 2^-ΔΔCt^ method [74] with *Eef2* (for cDNA) or input (for ChIP) as the normalizing control. Negative control for ChIP was a gene desert in chromosome 15 [75]. Primer sequences are available in S4 Table.

### Transcriptomic analysis

RNA from isolated hepatocytes and snap frozen liver tissues was extracted with the TRIzol reagent as above with alteration to the protocol. The aqueous phase after phase separation using chloroform was transferred to spin columns from PureLink RNA Mini Kit (12183025; Thermo Fisher Scientific) and washed with provided wash buffers. Treatment with rDNase (740963; Macherey-Nagel) was performed on column and eluted with provided RNase-free water. RNA fragmentation, library generation, and sequencing were performed as previously described [32]. The data from the RNA sequencing is shown in S1 Table and the raw data has been deposited at GEO under GSE159497 (WT and *Cdk1*^*Liv-/-*^ isolated hepatocytes and whole liver from 2-week-old mice).

### Basescope assay

Custom probes targeting *Cdk1* exon 3 were designed by ACDBio. Formalin-fixed liver sections were subjected to Basescope assay using the Basescope Detection Reagent Kit–RED (ACDBio) according to manufacturer’s protocol [76].

### Cytokine array

50mg of snap frozen liver tissue was homogenized using a dounce homogenizer in 500μL PBS supplemented with protease inhibitor cocktail (Sigma-Aldrich). 1μL of Triton X-100 was added and lysate was frozen at -80°C for at least 30 minutes. Lysates were then thawed and centrifuged at 13,000g for 10 minutes to remove any debris. Array was performed on the lysate using the Proteome Profiler Mouse Cytokine Array, Panel A (ARY006; R&D Systems) according to provided instructions, detection was done with 2mL of chemiluminescence substrate, and exposure to photographic film (Kodak), quantification was performed using ImageJ [77].

### Biological sample collection

Liver was harvested when mice reached the indicated timepoints and fixed in 10% neutral buffered formalin (NBF) for histology, frozen in Tissue-Tek OCT compound (Sakura Finetek) in liquid nitrogen-cooled isopentane for cryosections, or snap frozen in liquid nitrogen for RNA and protein extraction. Blood was collected by cardiac puncture, kept in lithium heparin-coated Microvette 500 LH (20.1345.100; Sarstedt), and analysed using the Comprehensive Diagnostic Profile rotor (500–0038; Abaxis) on the Vetscan VS2 (Abaxis) for liver function, or using the Hemavet 950 (Drew Scientific) for blood leukocyte counting. Embryonic day 14.5 (E14.5) hepatoblasts and postnatal day 14 (P14) hepatocytes were isolated using a previously published protocol [78].

### Protein extraction and immunoblotting

Protein extraction and immunoblotting were performed as previously described [32]. Mitochondria-cytoplasm fractionation of isolated hepatocytes was executed as detailed [79]. Antibodies used for immunoblotting include anti-CDK1 (1:1000; ab18; Abcam), anti-CDK2 (1:200; sc-6248; Santa Cruz Biotechnology), anti-CHK1 (1:400; sc-8408; Santa Cruz Biotechnology), anti-CHK1 pS345 (1:500; 2348; Cell Signaling Technology), anti-COX IV (1:500; 4844; Cell Signaling Technology), anti-CYC (1:1000; 4280; Cell Signaling Technology), anti-p21^*Cdkn1a*^ (1:200; sc-6246; Santa Cruz Biotechnology), anti-p53 (1:2000; #9282; Cell Signaling Technology), anti-p53 pS15 (1:2000; #9284; Cell Signaling Technology), anti-TUBB (1:5000; MRB-435P, Covance Antibody Products) and anti-HSP90 (1:5000; 610418; BD Biosciences). Quantification of blots was done using ImageJ software [77].

### Hematoxylin & eosin (H&E) and sirius red staining

Liver tissues were fixed in 10% NBF for 24 hours, dehydrated via ethanol baths and then embedded in paraffin blocks using a Leica EG1160 machine. Paraffin-embedded tissues were sectioned to 5μm thickness, adhered to Superfrost Plus slides (4951PLUS; Thermo Fisher Scientific), and dried overnight. Slides were de-paraffinized in xylene baths and re-hydrated in de-ionized water. H&E staining of re-hydrated slides was performed as before [80]. For Sirius Red staining, rehydrated sections were stained in Picro-Sirius Red stain (ab246832; Abcam) for 4 hours and washed in running water for 2 minutes. Stained slides were dehydrated in isopropanol baths and xylene before being mounted with Eukitt quick-hardening mounting medium (Sigma-Aldrich) and coverslip and allowed to dry overnight. Images were captured using the Olympus BX-61 upright microscope with 20x air lens or 40x oil lens. Quantification of Sirius Red-positive area was done using Fiji [81].

### Immunohistochemistry and immunofluorescence

Formalin-fixed liver sections from paraffin blocks were de-paraffinized. De-paraffinized slides were submersed in 1% hydrogen peroxide (in methanol) to block endogenous peroxidase activity before being rehydrated in deionized water. Antigen retrieval was then performed either using Proteinase K solution or trisodium citrate solution. For antigen retrieval in Proteinase K solution, slides were incubated with 20μg/mL Proteinase K in Tris-EDTA buffer (10mM Tris-HCl pH8.0, 1mM EDTA) at 37°C for 20 minutes. Antigen retrieval in trisodium citrate solution has been described previously [82]. Following antigen retrieval, slides were washed in PBS and blocked in 1% BSA in PBS supplemented with 0.1% Tween (0.1% PBS-T). Slides were then incubated with primary antibodies diluted in 0.1% BSA in 0.1% PBS-T for 1 hour at room temperature before washing in 0.1% PBS-T. For immunohistochemistry, slides were subsequently developed using the Dako DAB + Substrate Chromogen System (Agilent) according to manufacturer’s protocol, washed with running water, counterstained with 10% Harris Hematoxylin solution, washed with running water, and then mounted as above. Images were captured using an Olympus BX-61 microscope. Antibodies used for immunohistochemistry were anti-F4/80 (1:200; MCA497GA; Bio-Rad) and anti-PCNA (1:1000; #2586; Cell Signaling Technology). For immunofluorescence, antigen retrieval was done as above, blocking, primary and secondary antibody incubations, and image acquisition were performed as previously described [82]. Antibodies used for immunofluorescence were anti-histone H3 pS10 (1:200; 06–570; Merck Millipore) and anti-γH2AX (1:150; 05–636; Sigma-Aldrich).

### Flow cytometry

Pelleted isolated hepatocytes were resuspended in complete DMEM containing 0.5mg/mL propidium iodide solution (Sigma-Aldrich) to a density of about 1x10^6^ cells per mL. DNA profile was analysed using the BD LSRII (BD Biosciences) and FlowJo software.

### Statistical analysis

All experiments were performed with at least three biological replicates. Statistical analyses, aside from RNAseq data analysis, Mendelian genetic analysis and quantification of binuclear/mononuclear hepatocytes, were performed with unpaired T-test with Welch’s correction using GraphPad Prism version 6 (GraphPad Software). Mendelian genetic analysis was performed with chi-square test and quantification of binuclear/mononuclear hepatocytes was analysed with two-way ANOVA using GraphPad Prism. Statistical significance was considered when p-value<0.05 and shown as: p-value<0.05 (*); p-value<0.01 (**); p-value<0.001 (***).

For RNAseq data analysis, sequence reads were aligned using the STAR mapper [83] and fragments per kilobase million (FPKM) quantification was performed using the RSEM tool [84]. A standard data quality control procedure was performed on the sequence alignment results using FastQC, and we chose not to perform data normalization upon inspecting the equal distribution of FPKM values across the samples. To remove artefacts of statistical analysis in extremely low abundance genes, a small fudge factor (0.5) was added to all FPKM values and differential expression analysis done using two sample T-test. We adjusted p-values for multiple testing by calculating q-values [85], and selected all genes with q-value 0.1 or lower as significantly differentially expressed genes. Raw data for all plots are listed in S5 Table.

## Supporting information

S1 Fig *Cdk1*^*Liv-/-*^ hepatocytes are prone to apoptosis. (A) qPCR for *Bcl2l11* and *Bbc3* mRNA expression in P14 isolated hepatocytes (n = 3 per genotype). (B) Immunoblot of mitochondria-cytoplasm fractionated lysates from P14 isolated hepatocytes, probing for cytochrome c (CYC), using β-tubulin (TUBB) as cytoplasmic marker and COX IV as mitochondrial marker. (C) Quantification of cytoplasmic cytochrome c, normalizing to β-tubulin as loading control. (D) Quantification of sub-G1 population of cells from flow cytometry experiments (at least n = 5 per genotype).
(TIF)

S2 Fig (related to Fig 3). Detailed heat map of gene ontologies as has been described in Fig 3F. Color scale represents–log_10_(p-value).
(TIF)

S3 Fig (related to Fig 3). (A) Detailed heat map for list of cell cycle associated genes. For description see Fig 3G. Color scale represents Z-score. (B) Validation of selected cell cycle genes by qPCR in P14 isolated hepatocytes (n = 3 per genotype).
(TIF)

S4 Fig (related to Fig 3). Detailed heat map for list cytokinesis associated genes. Color scale represents Z-score.
(TIF)

S5 Fig (related to Fig 3). Detailed heat map for list of DNA damage associated genes. Color scale represents Z-score. For description see Fig 3H.
(TIF)

S6 Fig (related to Fig 4). Detailed heat map for list of fibrosis associated genes. Color scale represents Z-score.
(TIF)

S7 Fig (related to Fig 6 and Fig 7). **Gating strategy for flow cytometry analysis of DNA content.** Propidium iodide-stained isolated hepatocytes were first gated for live cells in an FSC-A vs SSC-A plot, after which live cells were gated for singlets in an FSC-A vs FSC-H plot.
(TIF)

S1 Table List of genes detected by RNAseq in wild-type (WT) and *Cdk1*^*Liv-/-*^ isolated hepatocytes and whole liver. (XLSX)

S2 Table List of gene ontologies enriched among statistically significant differentially expressed genes from the RNAseq data. (XLSX)

S3 Table Distribution of *Cdk2* genotype in a *Cdk1*^*Liv-/-*^ background, with numbers and percentage (%) for each genotype. Deviation from Mendelian genetic ratio was calculated using chi-square test.
(XLSX)

S4 Table List of primer sequences used for qPCR and ChIP-qPCR. (XLSX)

S5 Table In separate sheets, the excel spreadsheet contains the numerical data for Figs 1A, 1D, 1F, 1H, 1I, 2A, 2B, 2D, 2F, 2G, 2I, 2J, 2K, 2L, 3A, 3B, 3C, 4B, 4C, 4E, 5B, 5D, 5E, 5F, 6B, 6E, 7B, 7E, 7F, 7G, 7I, 7J, 7K, S1A, S1C, S1D and S3B. (XLSX)
