# Supplementary figures and images for: Loss of hepatocyte cell division leads to liver inflammation and fibrosis
Source: PLoS Genet. 2020 Nov 4;16(11):e1009084. doi: 10.1371/journal.pgen.1009084 (PMC7641358; doi:10.1371/journal.pgen.1009084)

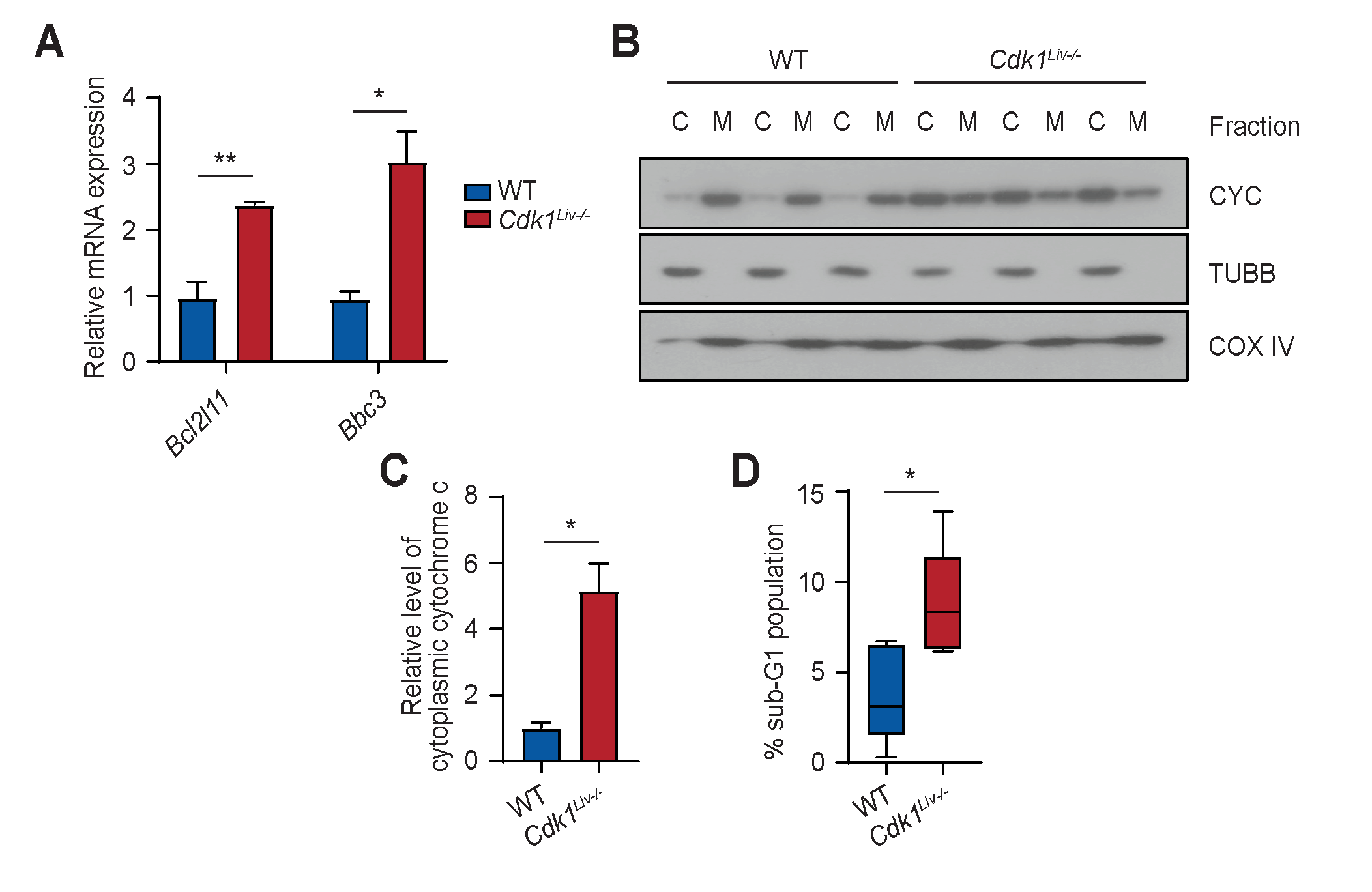

Supplement: S1 Fig — (A) qPCR for Bcl2l11 and Bbc3 mRNA expression in P14 isolated hepatocytes (n = 3 per genotype). (B) Immunoblot of mitochondria-cytoplasm fractionated lysates from P14 isolated hepatocytes, probing for cytochrome c (CYC), using β-tubulin (TUBB) as cytoplasmic marker and COX IV as mitochondrial marker. (C) Quantification of cytoplasmic cytochrome c, normalizing to β-tubulin as loading control. (D) Quantification of sub-G1 population of cells from flow cytometry experiments (at least n = 5 per genotype). (TIF) [file pgen.1009084.s001.tif]

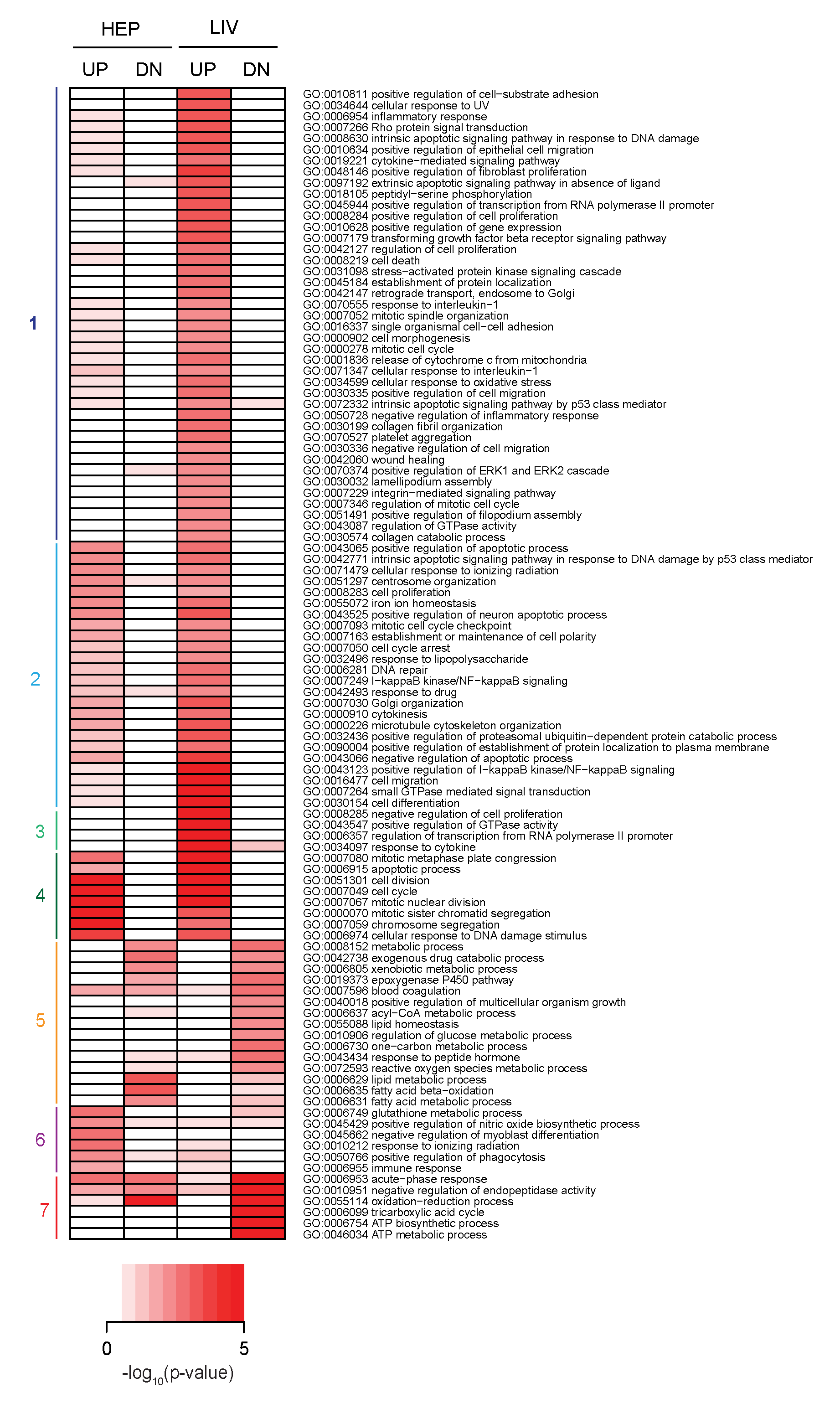

Supplement: S2 Fig — Detailed heat map of gene ontologies as has been described in Fig 3F. Color scale represents–log10(p-value). (TIF) [file pgen.1009084.s002.tif]

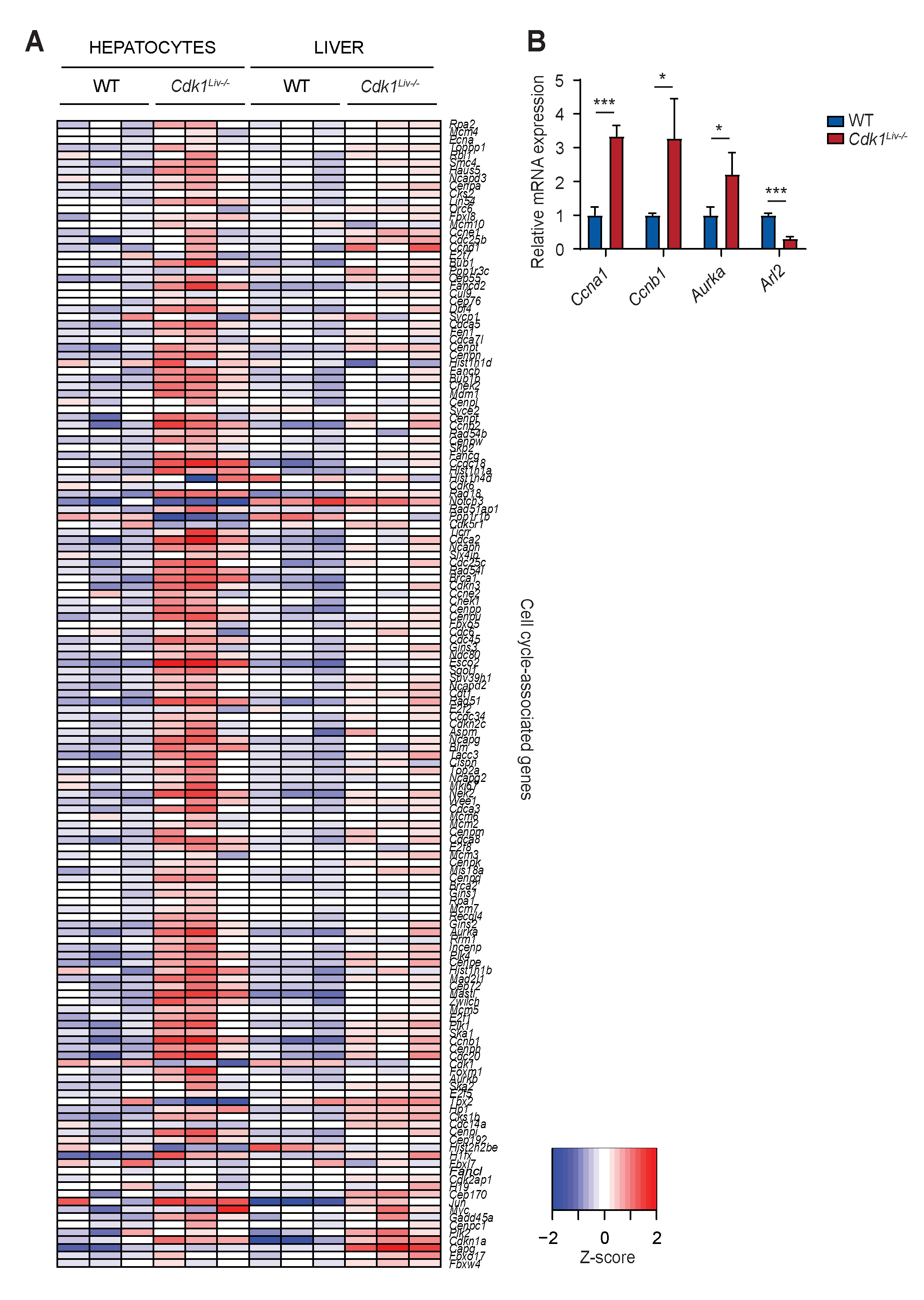

Supplement: S3 Fig — (A) Detailed heat map for list of cell cycle associated genes. For description see Fig 3G. Color scale represents Z-score. (B) Validation of selected cell cycle genes by qPCR in P14 isolated hepatocytes (n = 3 per genotype). (TIF) [file pgen.1009084.s003.tif]

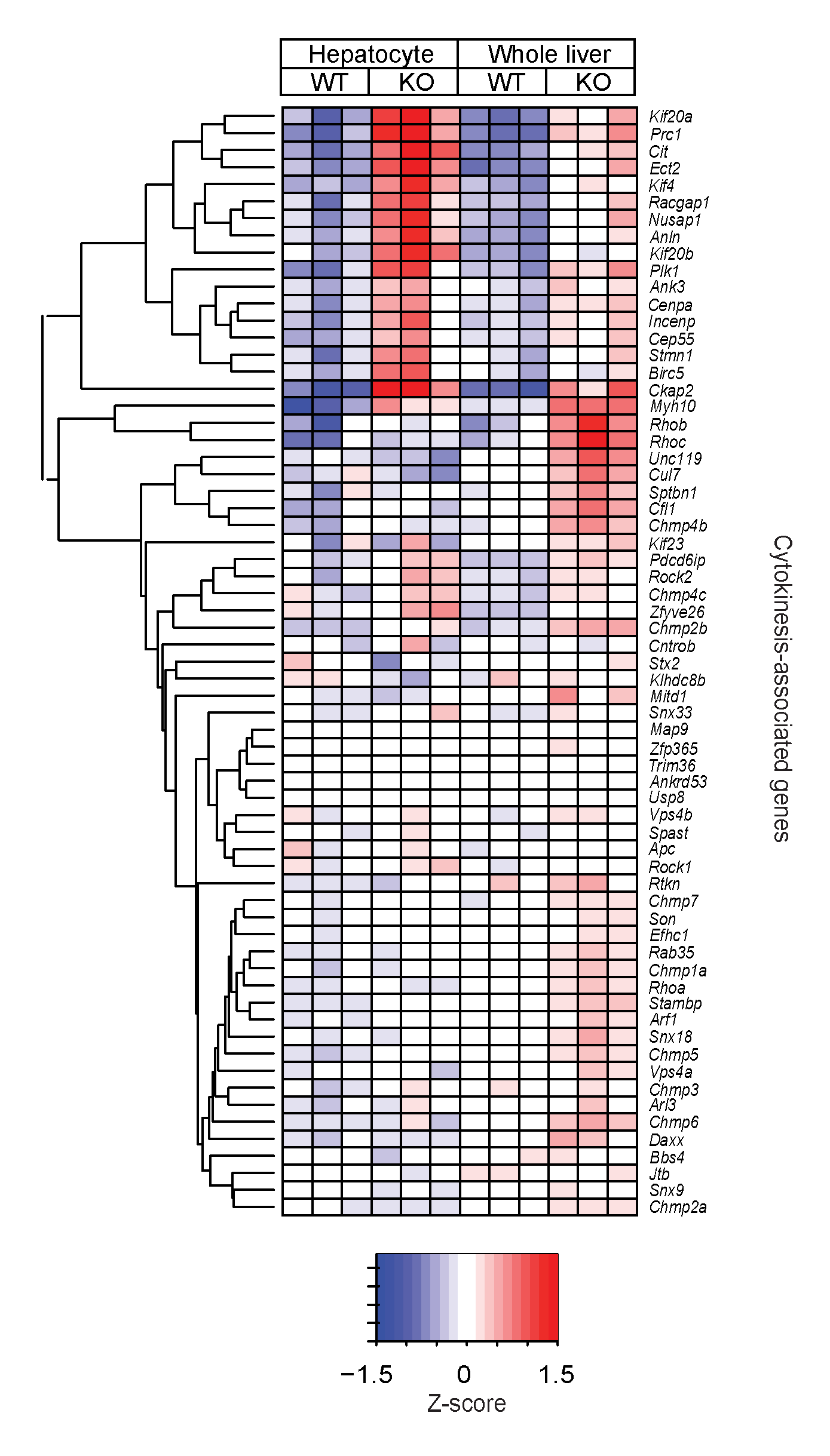

Supplement: S4 Fig — Detailed heat map for list cytokinesis associated genes. Color scale represents Z-score. (TIF) [file pgen.1009084.s004.tif]

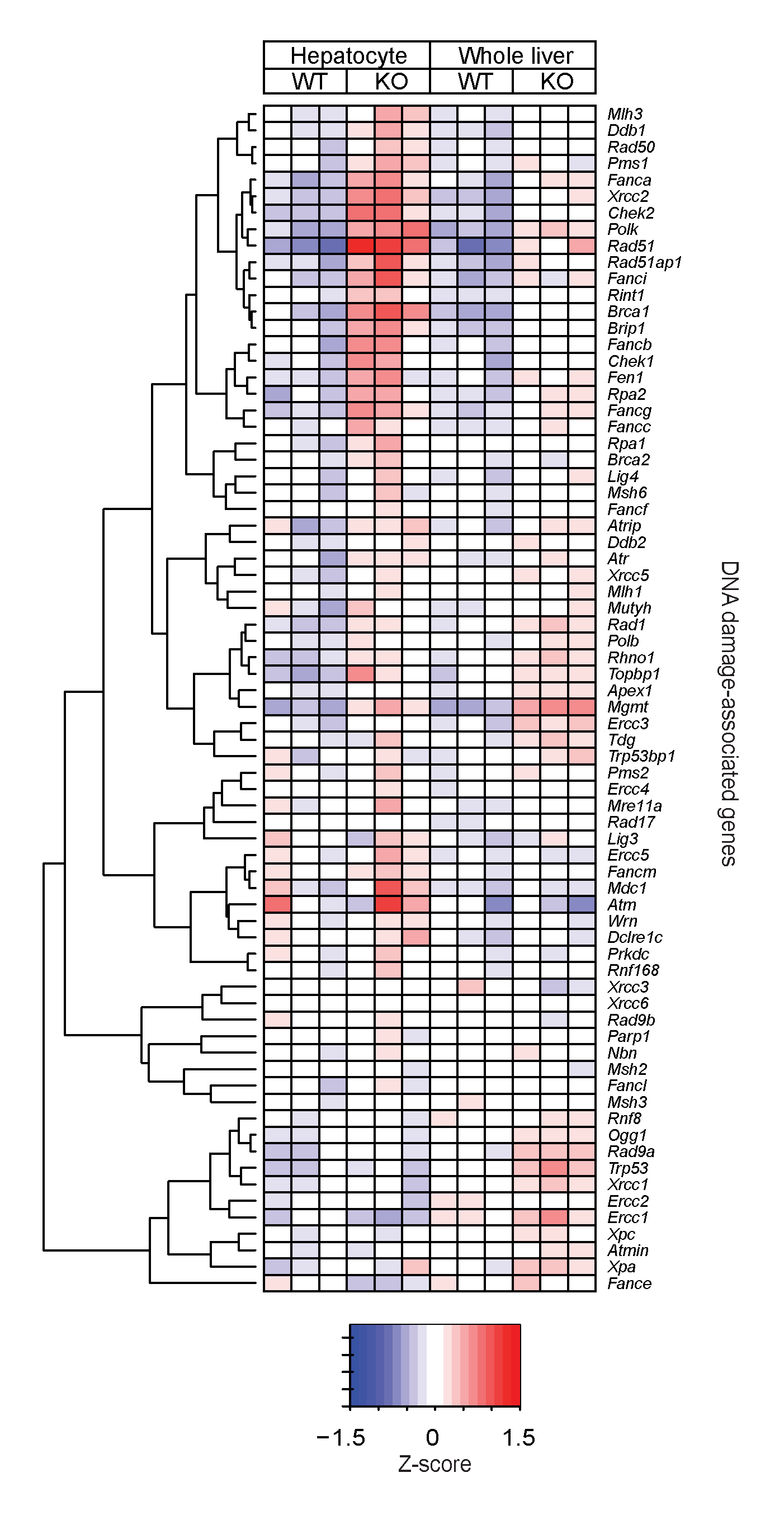

Supplement: S5 Fig — Detailed heat map for list of DNA damage associated genes. Color scale represents Z-score. For description see Fig 3H. (TIF) [file pgen.1009084.s005.tif]

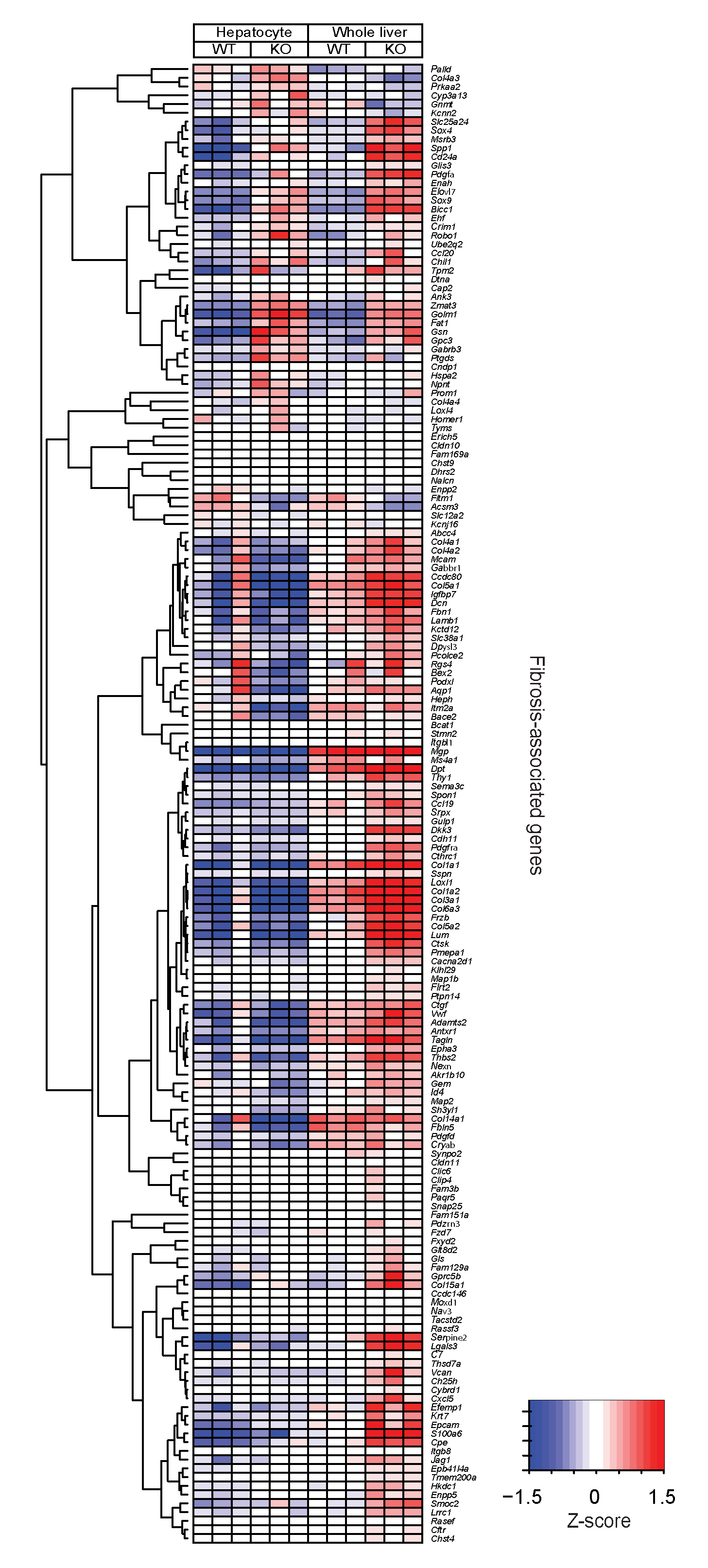

Supplement: S6 Fig — Detailed heat map for list of fibrosis associated genes. Color scale represents Z-score. (TIF) [file pgen.1009084.s006.tif]

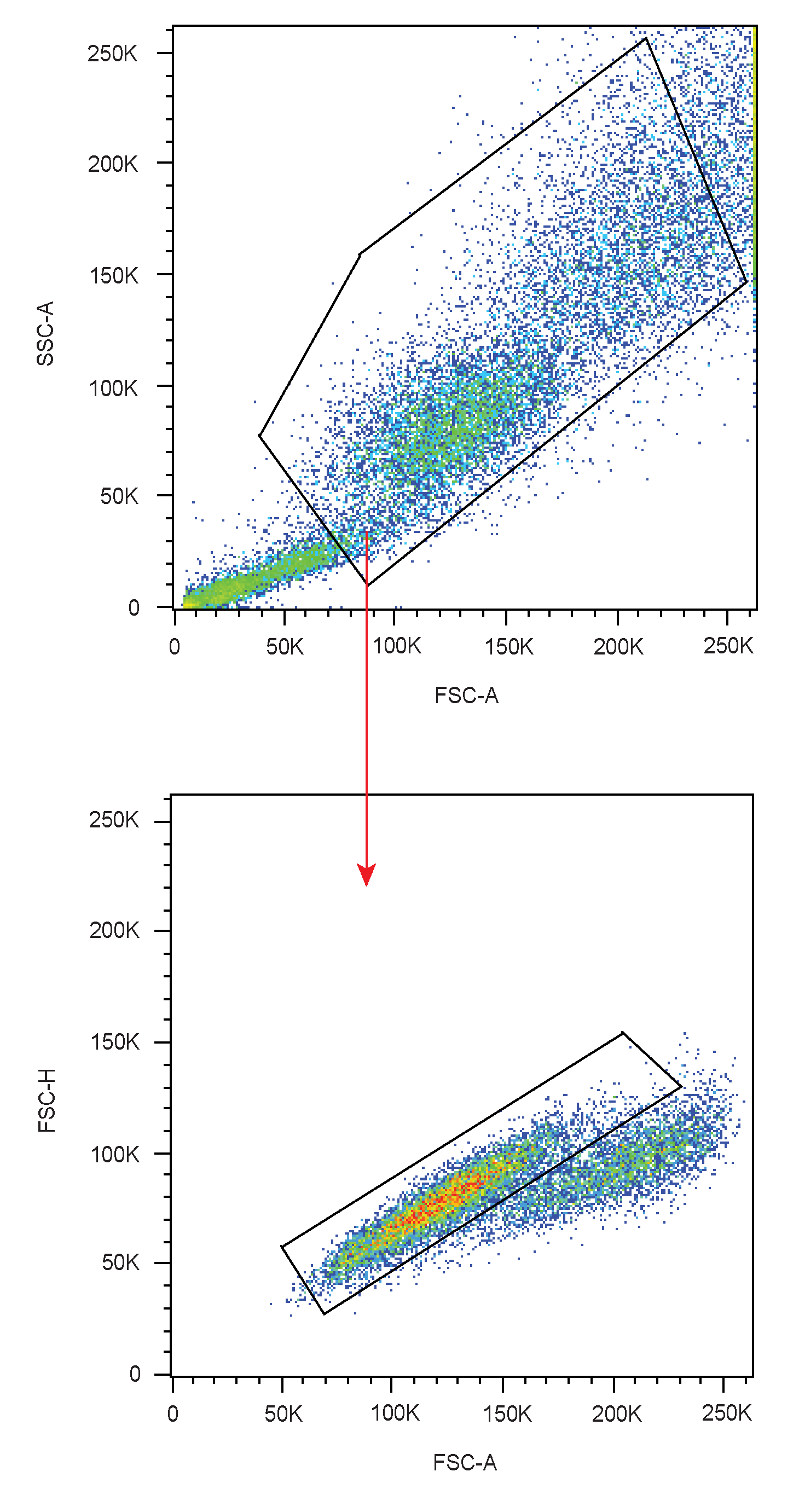

Supplement: S7 Fig — Gating strategy for flow cytometry analysis of DNA content. Propidium iodide-stained isolated hepatocytes were first gated for live cells in an FSC-A vs SSC-A plot, after which live cells were gated for singlets in an FSC-A vs FSC-H plot. (TIF) [file pgen.1009084.s007.tif]
